# ROS Production Is Essential for the Apoptotic Function of E2F1 in Pheochromocytoma and Neuroblastoma Cell Lines

**DOI:** 10.1371/journal.pone.0051544

**Published:** 2012-12-12

**Authors:** Lilia Espada, Nathalie Meo-Evoli, Patricia Sancho, Sebastian Real, Isabel Fabregat, Santiago Ambrosio, Albert Tauler

**Affiliations:** 1 Departament de Bioquímica i Biologia Molecular, Facultat de Farmàcia. Universitat de Barcelona, Barcelona, Catalunya, Spain; 2 Cancer and Metabolism Group, Bellvitge Biomedical Research Institute (IDIBELL), L’Hospitalet de Llobregat, Catalunya, Spain; 3 Biological Clues of the Invasive and Metastatic Phenotype Group, Bellvitge Biomedical Research Institute (IDIBELL), L’Hospitalet de Llobregat, Catalunya, Spain; 4 Unitat de Bioquímica, Departament de Ciències Fisiològiques II, Facultat de Medicina, Campus Universitaride Bellvitge - IDIBELL, Universitat de Barcelona, L’Hospitalet de Llobregat, Catalunya, Spain; Universitat de Lleida - IRBLLEIDA, Spain

## Abstract

In this study we demonstrate that accumulation of reactive oxygen species (ROS) is essential for E2F1 mediated apoptosis in ER-E2F1 PC12 pheochromocytoma, and SH-SY5Y and SK-N-JD neuroblastoma stable cell lines. In these cells, the ER-E2F1 fusion protein is expressed in the cytosol; the addition of 4-hydroxytamoxifen (OHT) induces its translocation to the nucleus and activation of E2F1target genes. Previously we demonstrated that, in ER-E2F1 PC12 cells, OHT treatment induced apoptosis through activation of caspase-3. Here we show that caspase-8 activity did not change upon treatment with OHT. Moreover, over-expression of Bcl-xL arrested OHT-induced apoptosis; by contrast, over-expression of c-FLIP, did not have any effect on OHT-induced apoptosis. OHT addition induces BimL expression, its translocation to mitochondria and activation of Bax, which is paralleled by diminished mitochondrial enrichment of Bcl-xL. Treatment with a Bax-inhibitory peptide reduced OHT-induced apoptosis. These results point out the essential role of mitochondria on the apoptotic process driven by E2F1. ROS accumulation followed E2F1 induction and treatment with the antioxidant *N*-acetylcysteine, inhibited E2F1-induced Bax translocation to mitochondria and subsequent apoptosis. The role of ROS in mediating OHT-induced apoptosis was also studied in two neuroblastoma cell lines, SH-SY5Y and SK-N-JD. In SH-SY5Y cells, activation of E2F1 by the addition of OHT induced ROS production and apoptosis, whereas over-expression of E2F1 in SK-N-JD cells failed to induce either response. Transcriptional profiling revealed that many of the genes responsible for scavenging ROS were down-regulated following E2F1-induction in SH-SY5Y, but not in SK-N-JD cells. Finally, inhibition of GSK3β blocked ROS production, Bax activation and the down regulation of ROS scavenging genes. These findings provide an explanation for the apparent contradictory role of E2F1 as an apoptotic agent versus a cell cycle activator.

## Introduction

Increased expression of the E2F1 transcription factor is found in high risk neuronal tumors and has been proposed as a factor responsible for the initiation step of tumorigenesis [1]. E2F1 plays an essential role in G1/S transition and directly induces the transcription and expression of N-myc in neuroblastoma cell lines [2]. Deregulation of N-myc is thought to play an important role in tumor progression, with N-myc amplification strongly associated with a poor prognosis [3]. Although it is widely accepted that E2F1 acts as a potent activator of cell cycle progression, it is known to induce apoptosis under specific conditions [4]. The final decision of whether E2F1 functions to mediate cell cycle progression or apoptosis is a consequence of the integration of cellular and environmental signals. A greater knowledge of the molecular mechanisms by which E2F1 drives apoptosis would enhance our understanding of its role in neuronal tumorigenesis and its potential as a therapeutic target.

In mammals, the E2F family of transcription factors is composed of eight members. E2F1-3 form heterodimers with DP proteins and act primarily as transcriptional activators, whereas E2F-4-8 primarily function as transcriptional repressors. Activation of E2F results in the transcriptional modulation of specific genes involved in DNA replication, development, differentiation and apoptosis [5]. It has been suggested that neuronal apoptosis can also result from de-repression, not trans-activation of E2F-responsive genes [6]. Among the eight members of the E2F family described above, E2F1 is unique in its ability to induce apoptosis [4]. Over-expression of E2F1 promotes apoptosis in several neuronal populations and is required for neuronal death induced by a variety of stimuli [7]. Neurons lacking E2F1 (derived from E2F1^−/−^ mice) are largely protected from apoptosis induced by growth factor withdrawal, staurosporine, β-amyloid dopamine, suggesting that E2F1 protein is essential in this response [7]–[9].

Despite numerous studies indicating the participation of E2F1 in neuronal apoptosis, the mechanism through which E2F1 induces apoptosis is not well understood. E2F1 has been demonstrated to participate in both, extrinsic and intrinsic apoptotic pathways. In lung adenocarcinoma cells, E2F1 induces the extrinsic apoptosis by down-regulating the expression of FLICE-inhibitory protein short, leading to caspase-8 activation by the death-inducing signaling complex [10]. However, others have demonstrated that E2F1 promotes the transcription of Bid and caspase-8, molecules that link death receptor signaling to the activation of apoptotic mechanism in mitochondria [11]. Consistent with the intrinsic pathway, in cultured cerebellar granule neurons, cells lacking E2F1 were less susceptible to Fas-mediated apoptosis in comparison to their wild type counterpart [12].

The activation of intrinsic apoptotic pathway by E2F1 occurs via both p53-dependent and p53-independent mechanisms. In the first step of the p53-dependent pathway, E2F1 activates the transcription of the tumor suppressor ARF. ARF drives the accumulation of p53 protein *via* the direct association and inhibition of the p53-ligase, Mdm2. E2F1 can also signal apoptosis independently of p53 by directly activating the transcription of p53 family member, p73. In addition, Bcl-2 family members have been found to be direct targets of E2F1 such as Bak, Bax, PUMA, Noxa, Bim, HrK, and Apaf-1, whereas E2F1 represses the transcription of anti-apoptotic protein Mcl1 [13]. Finally, alternative models suggest that E2F1 can potentiate apoptosis through generation of reactive oxygen species (ROS), either by inhibiting NF-κB or by transcriptional upregulation of NADPH oxidase NOX4 [14], [15].

We have shown previously that over-expression of E2F1 induces apoptosis in naive as well as differentiated pheochromocytoma cell line PC12 [16]. Here we show that the E2F1-induced ROS production is required for the apoptotic functions of E2F1. These findings provide an explanation for the apparent contradictory role of E2F1 as an apoptotic agent versus a cycle activator.

## Materials and Methods

### Cell Culture, Transfection and Plasmids

ER-E2F1 PC12 rat pheochromocytoma stable cells were cultured in DMEM high glucose media without pyruvate and supplemented with 12% of heat-inactivated serum (6% fetal bovine serum and 6% horse serum)(all from Gibco, UK) in the presence of 0,5 mg/ml of the selective agent geneticin (Sigma-Aldrich, USA). The neuroblastoma ER-E2F1 stable cell lines SH-SY5Y and SK-N-JD were grown in DMEM containing 10% fetal bovine serum and RPMI 10% fetal bovine serum plus 1% L-Glutamine, respectively [16]. For proper cell attachment, all experiments were performed in plates coated with 0,1 mg/ml poly-DL-ornithine for PC12 cells and with fibronectin at 4,5 µg/ml for neuroblastoma cells (both from Sigma-Aldrich, USA). ER-E2F1 translocation to the nuclei was achieved by incubating cells with 400 nM of 4-hydroxytamoxifen (OHT) (Calbiochem USA). The set of inhibitors used in this study was N-Acetyl-Cysteine (NAC) at 1 mM, Diphenyleneiodonium Chloride (DPI) at 500 nM (both from Sigma-Aldrich, USA), Bax-inhibiting peptide V5 (Calbiochem USA) and LiCl at 40 mM (Merck, USA). For the latter a pre-incubation period of 30 minutes was performed, and for all treatment conditions the control cells were treated with dimethylsulfoxide (DMSO). DMSO concentrations did not exceed 0,05%.

**Figure 1:**
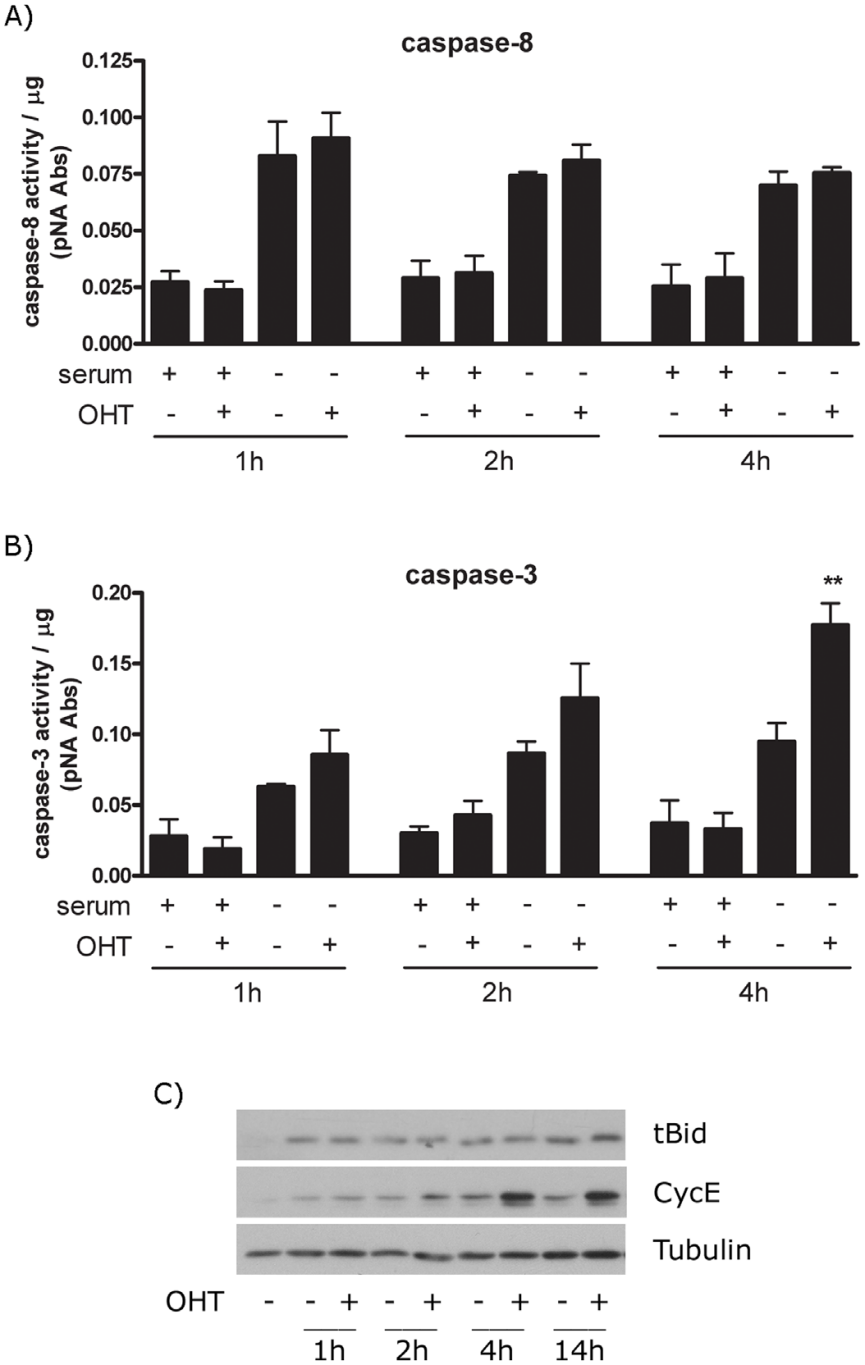
The extrinsic apoptotic pathway is not involved in E2F1-induced apoptosis. (A, B) Stable ER-E2F1 PC12 cells were treated (+) or not treated (−) with OHT in the presence or absence of serum for the indicated hours. Cells were collected and lysed, and caspase-8 (A) or caspase-3 (B) activities were analysed using the *p*NA colorimetric assay as indicated in Material and Methods. (C) Stable ER-E2F1 PC12 cells were treated with OHT at the indicated times. Mitochondrial fractions were obtained and expression of the indicated proteins was determined by Western blot. Results are presented as Mean ± SEM, for n = 3. Student’s *t*-test value of **p<0,01 was considered statistically significant.

**Figure 2:**
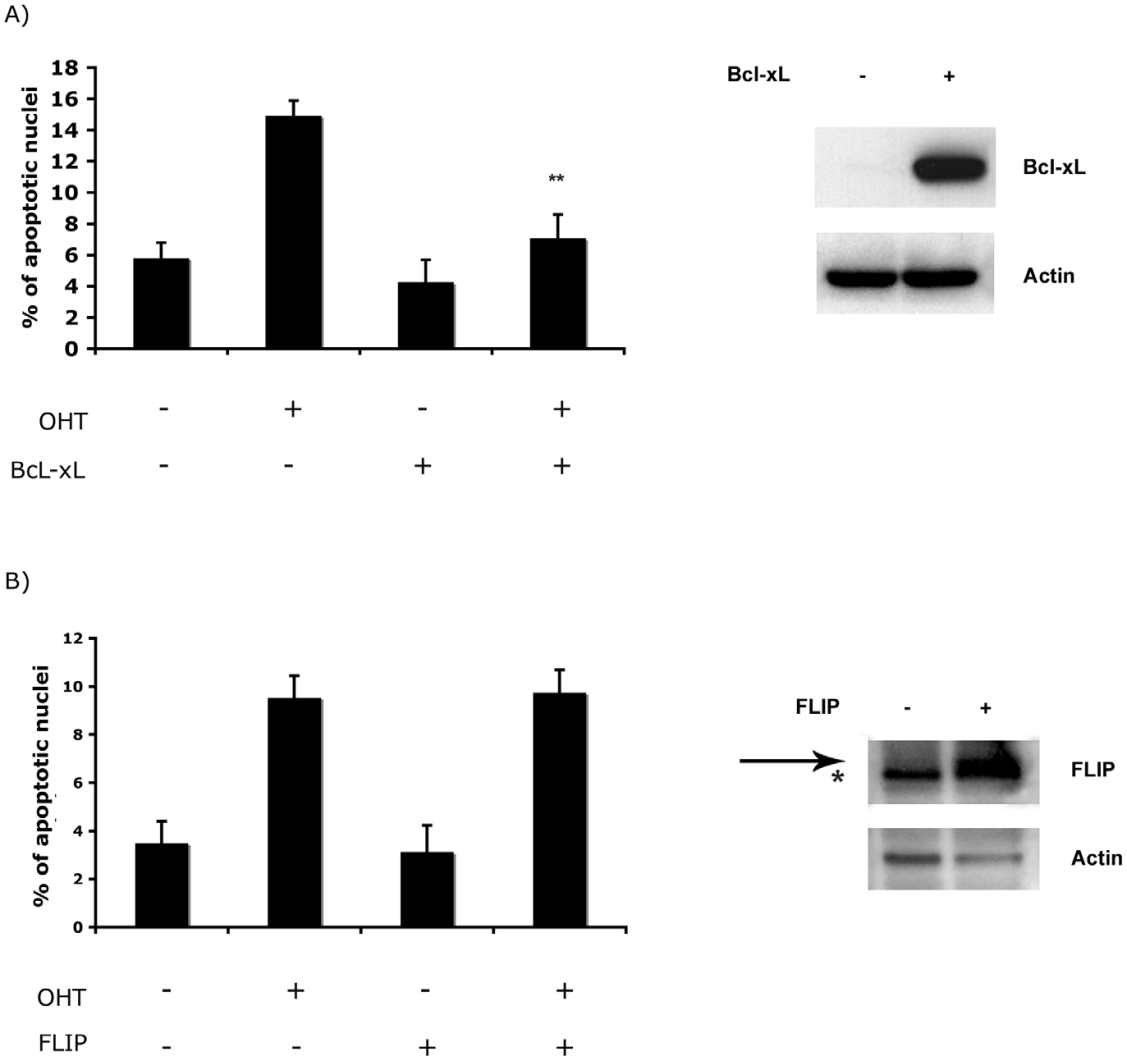
Effect of over-expression of Bcl-xL and cFLIP in E2F1-induced apoptosis. Stable ER-E2F1 PC12 cells were transfected or not with Bcl-xL (A) or cFLIP (B) expression plasmids and treated (+) or not treated (−) with OHT. At 48 hours, apoptotic cells were identified by TUNEL and counted against 100 nuclei identified by Hoescht staining in randomly taken photographs. Results are presented as Mean ± SEM, for n = 3. Statistically significant differences were obtained comparing with cells untransfected. Student’s *t*-test value of **p<0,01 was considered statistically significant. Expression of the transfected proteins were measured by Western Blot analysis and shown on the right of the figures. HA antibody was used for Bcl-xL detection and FLAG for FLIP. Arrow indicates FLIP position and asterisk a non specific protein.

### Caspase-3 and -8 Assay

Caspase activities were measured by colorimetric assays assessing the cleavage of Ac-DVED-*p*NA substrate for caspase-3; and of Ac-IETD-*p*NA for caspase-8 (both from BD Biosciences, USA). Cells treated for the indicated times were washed once with ice-cold PBS and lysed on ice with 100–150 µl of ice-cold caspase lysis buffer (0,1% Triton X100, 150 mM NaCl, 50 mM HEPES pH7.4, 0,1 mM EDTA, 1 mM dithiothreitol, 1 mM DTT). The lysate was incubated in constant agitation for 30 min at 4°C, and finally centrifuged at maximum speed for 5 min at 4°C. After determination of protein content samples containing 30 µg of total cell lysate (caspase-3) or 100 µg (caspase-8) were assembled in duplicate in 96-well plates with freshly-prepared reaction buffer 2x (caspase lysis buffer supplemented with 20% glycerol and 20 mM DTT), and 100 µM of the adequate *p*NA colorimetric substrate was added to each well. The reaction mixture was incubated protected from light for 2 h at 37°C. Cleavage of the substrate by caspases releases *p*NA, detected by absorbance at 405 nm with a microplate reader. Caspase activity results are expressed as units of pNA absorbance per µg of protein.

**Figure 3:**
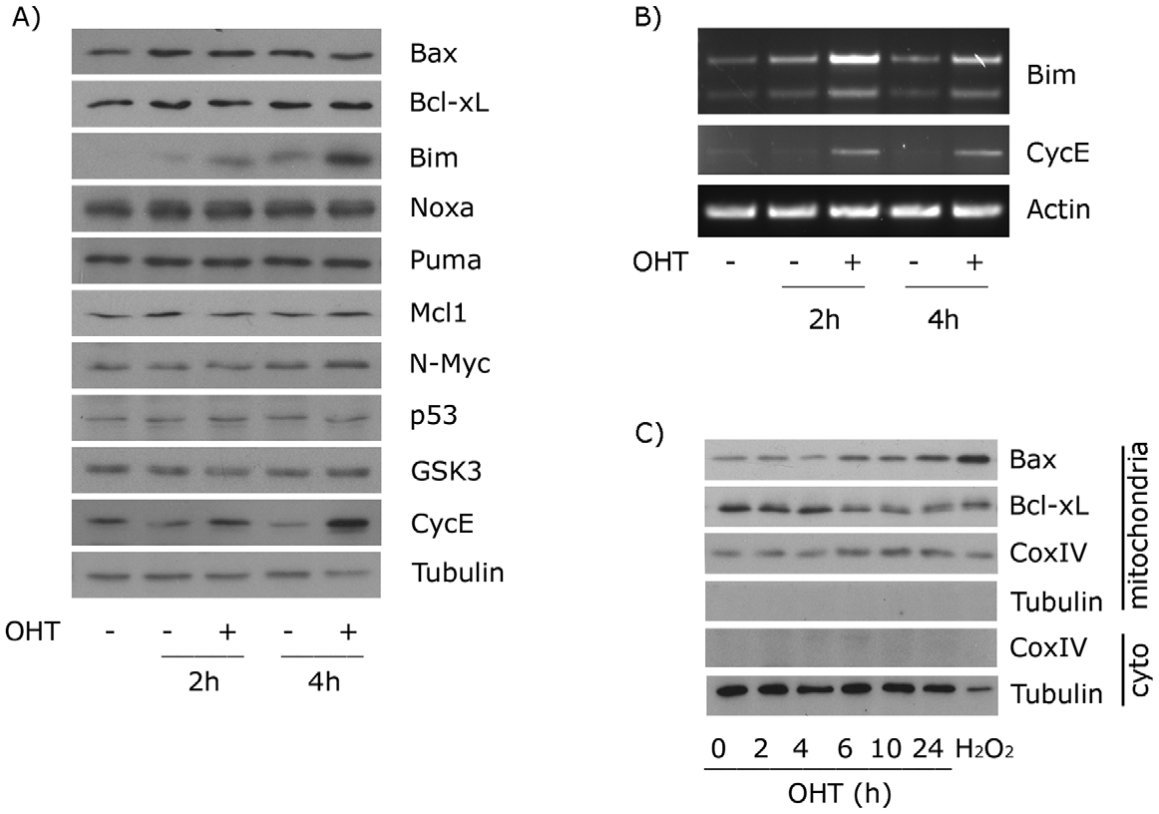
Expression analysis of reported E2F1-target genes in ER-E2F1 PC12 cells. (A) Serum-deprived cells were treated (+) or not treated (−) with OHT for the indicated hours. Expression of the indicated proteins was determined by Western Blot analysis. (B) Total mRNA was extracted and the levels of indicated transcripts were analyzed by RT-PCR. (C) ER-E2F1 PC12 cells were serum deprived and treated with OHT for the indicated hours, or with 400 µM of H_2_O_2_ for 6 hours. Mitochondrial fractions and cytoplasmic (cyto) fractions were obtained and analyzed for the indicated protein contents by Western blot analysis.

### TUNEL Assay

PC12 cells were grown and treated in poly-DL-ornithine coated 6 well plates (1×10^5^ cells per well) or 24 well plates (2×10^4^ cells per well). Cells were primary fixed by addition of 8% paraformaldehyde to the growth media for 30 min at room temperature. All media was then removed and cells were fixed with 4% paraformaldehyde for another 30 min at room temperature. After washing with PBS and PBS-T (PBS +0,3% TritonX-100), cells were blocked for 30 min with a 2% BSA, 0,3% TritonX-100 solution in PBS. They were washed again with PBS-T and incubated in reaction mixture for 2 hours at 37°C. Wells were isolated with parafilm to prevent them from drying and plates protected with aluminium foil paper to protect them from light. The composition of reaction mixture for 50 µl was as follows: 0.5 µl Terminal Transferase, 10 µl TdT Reaction Buffer (5×), 5 µl CoCl_2_ 25 mM (all from Roche, Germany), 0.5 µl ChromaTide Bodipy FL-14-dUTP (Molecular Probes, USA) and 1.5 µl Triton X-100 10% in H_2_O. For nuclear staining, cells were then incubated with 2 µg/µl Hoechst 33258 (Molecular Probes, USA) in PBT for 30 min at room temperature. Wells were washed in PBT and PBS and mounted in slow-fade light anti-fade solution. Cells were visualized under fluorescence microscopy. Each random field of cells was visualized for total nuclei counting (345/478 nm for Hoechst 33258 fluorescence), and for apoptotic nuclei counting (505/515 nm for Bodipy-FL fluorescence). Results were expressed as an apoptotic index defined as the number of apoptotic cells divided by number of total cells.

**Figure 4:**
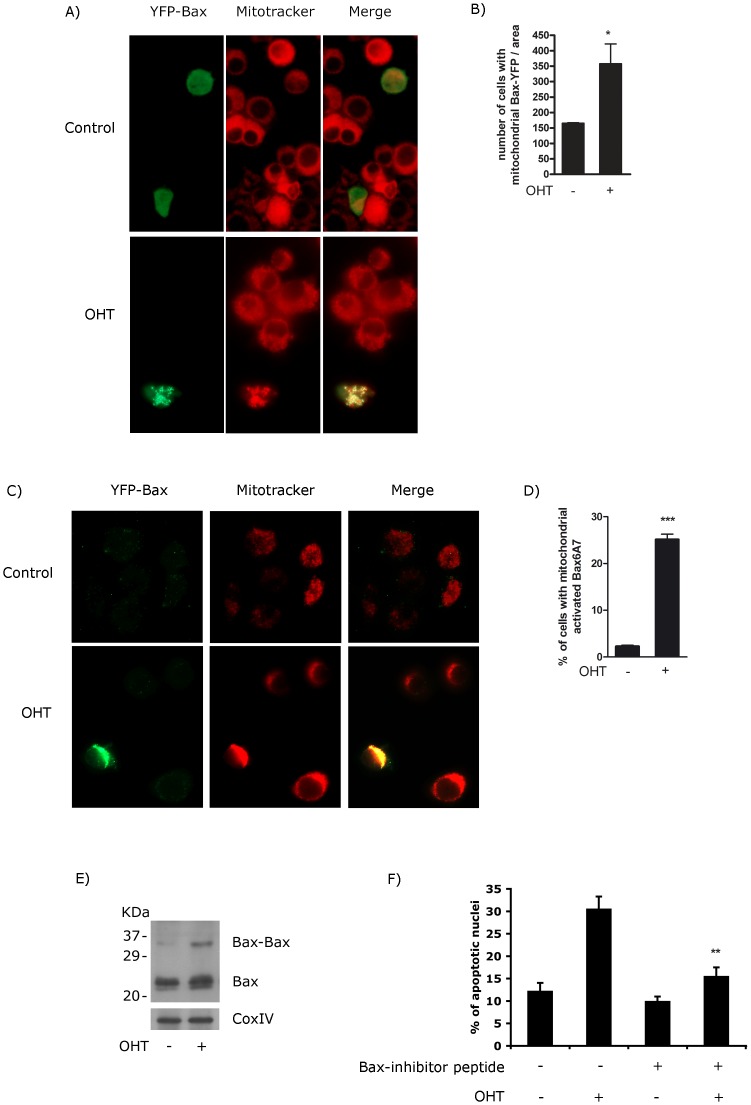
E2F1 induces Bax activation, dimerization and translocation to the mitochondria. (A) PC12 ER-E2F1 cells were transiently transfected with an YFP-Bax plasmid, serum-deprived and treated with (OHT) or without (control) OHT for 3 hours. YFP-Bax localization was achieved by immunofluorescence as indicated in Material and Methods. Red signal represents the mitochondrial staining with MitoTracker and green signal represents Bax-YFP localization. (B) Quantification of the punctuated mitochondrial clusters of YFP-Bax from the experiments described in A. (C) PC12 ER-E2F1 cells were serum-deprived and treated with (OHT) or without (control) OHT for 3 hours and active Bax was detected by immunofluorescence using anti-Bax 6A7 clone antibody. Red signal represents the mitochondrial staining with MitoTracker and green signal represents active Bax. (D) Quantification of the positive anti-BAx 6A7 from experiments described in C. (E) PC12 ER-E2F1 cells were treated with OHT at the indicated times and harvested and the mitochondria fraction was isolated. Crosslinking reactions were performed on the mitochondrial pellets as indicated in Materials and Methods. Samples were subjected to Western blot analysis and probed with anti-Bax antibody. (F) PC12 ER-E2F1 cells were treated with 50 µM of Bax-inhibiting peptide in the presence or in the absence of OHT. At 72 hours, apoptotic cells were identified by TUNEL and counted against 100 nuclei identified by Hoescht staining in randomly taken photographs. Results are presented as Mean ± SEM, for n = 3. Statistically significant differences were obtained comparing with cells untreated with Bax-inhibiting peptide. Student’s *t*-test value of **p<0,01 was considered statistically significant.

### Western Blot

Cells were harvested with growth media, and washed once with ice-cold PBS. After centrifugation, the cell pellet was resuspended and incubated for 30 min at 4°C in lysis buffer (20 mM Tris-HCl pH 8.0, 10 mM EDTA, 2,5 mM MgCl_2_, 1% Triton X100 with a supplement of 1 mM DTT and 1/100 dilution of phosphatase and protease inhibitor cocktail (Sigma Aldrich, USA). Equal amounts of protein lysate were subjected to 12% sodium dodecylsulphate (SDS) polyacrylamide gel electrophoresis and electrophoretically transferred to PVDF membranes. The membranes were then blocked with 5% non-fat milk solution in TBS-T (TBS with 0,1% Tween20) for 1 hour at room temperature and immunoblotted. The primary antibodies used were the following: CycE, Bax, N-Myc, p53, GSK3β, ΦΛΑG tag (Santa Cruz Biotechnology, USA); Bim, Noxa, Puma, Mcl-1 (Cell Signaling, USA); tBid (R&D Systems, USA); Bcl-xL (BD Transduction Laboratory, USA); Tubulin (Calbiochem, Germany); MnSOD (Upstate, USA) and HA Hemagglutinin Tag (Sigma, USA). Both incubations with primary and secondary horseradish peroxidase-conjugated antibodies (Santa Cruz Biotechnology, USA) were performed in 5% non-fat milk TBS-T solution for 2 h and 1 h, respectively. Blots were developed using an Enhanced Chemiluminescence kit (Amersham, UK).

**Figure 5:**
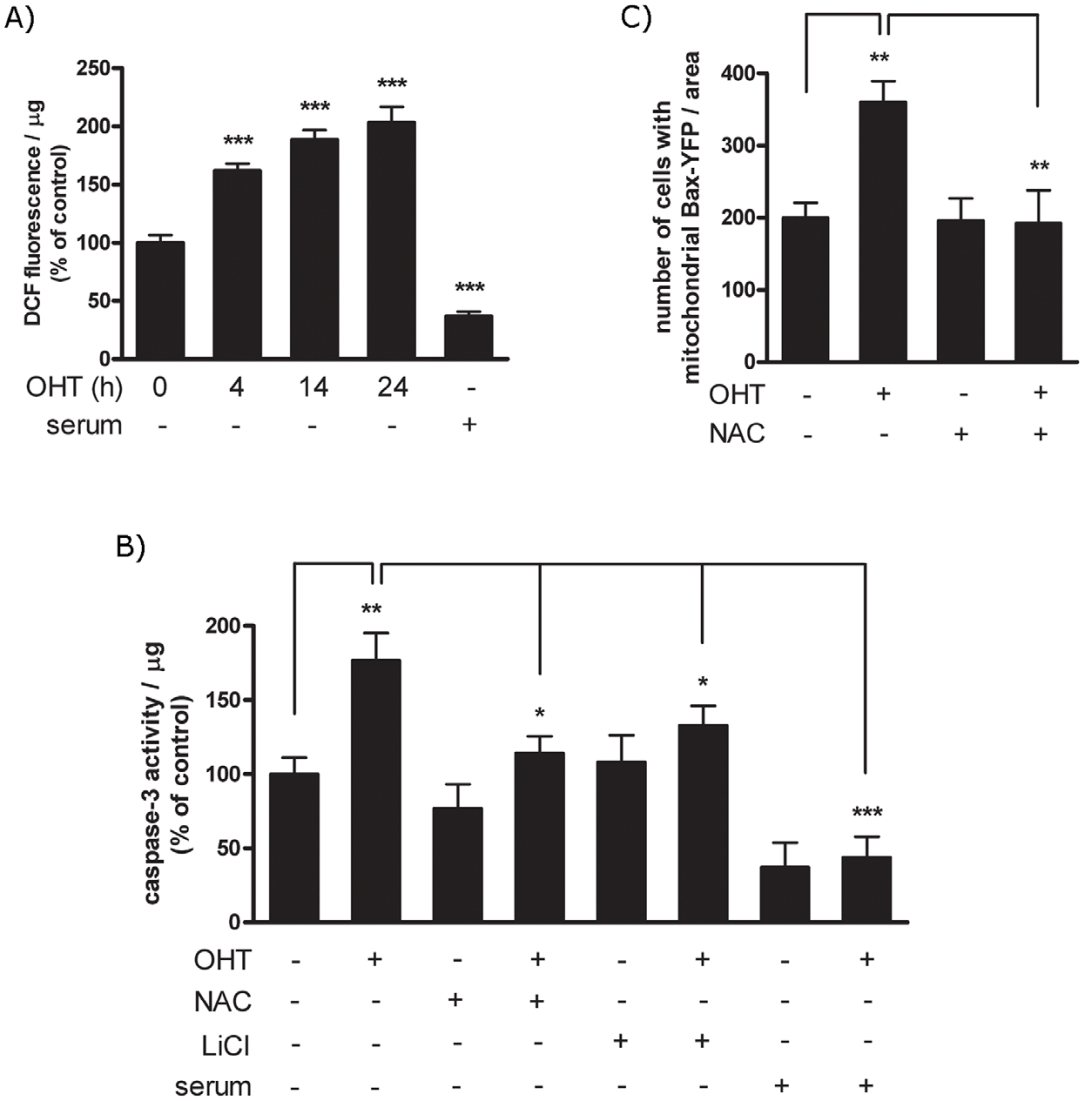
E2F1 enhanced ROS production is essential for the apoptotic function. (A) PC12 ER-E2F1 cells were serum deprived and treated with OHT for the indicated hours. ROS levels were analysed by using the oxidation-sensitive fluorescent probe H2DCFDA as described in Material and Methods. Results are presented as Mean ± SEM, for n = 6 (B) Stable ER-E2F1 PC12 cells were treated with (+) or without OHT in the presence (+) or in the absence (−) of 1 mM NAC, or in the presence (+) or in the absence of 40 mM LiCl or serum for 4 hours. After cell collection and lysis, caspase-3 activity of cell extracts was analysed by using the *p*NA colorimetric assay as indicated in Material and Methods. Results are presented as Mean ± SEM, for n = 3. (C) ER-E2F1 cells were transiently transfected with YFP-Bax, treated for 3 hours with (+) or without (−) OHT in the presence (+) or absence (−) of 1 mM NAC, and the punctuated mitochondrial clusters of YFP-Bax were quantified by immunostaining analysis. Results are presented as Mean ± SEM, for n = 3. In all of the figures, data are compared as indicated individually. Student’s *t*-test values of *p<0,05, **p<0,01 and ***p<0,0001 were considered statistically significant.

### PCR

Following treatment, total RNA was extracted using Ultraspec Total RNA Isolation from Tissues/Cells (Biotecx USA). 1 µg of total RNA was subjected to reverse transcription and the resulting cDNA samples were used in PCR amplification using KapaTaq Polymerase (KapaBiosystems, USA). The sequences of the PCR primers used were as followed: Bim, 5′-GGCCTGGGGCCCCTACCTCCCT-3′ (forward), and 5′-CCGCCGCAGCTCCTGTGCGAT-3′ (reverse); Cyc E, 5′-TCAGACCGCCCAGAGCC TCC-3′ (forward), and 5′-CCCCGGAGCAAGCACCATCA-3′ (reverse); and Actin, 5′-CAGAGCAAGCGAGGCATCCTG-3′ (forward), and 5′-GTTGAAGGTCTCAAACAT GATC-3′ (reverse).

**Figure 6:**
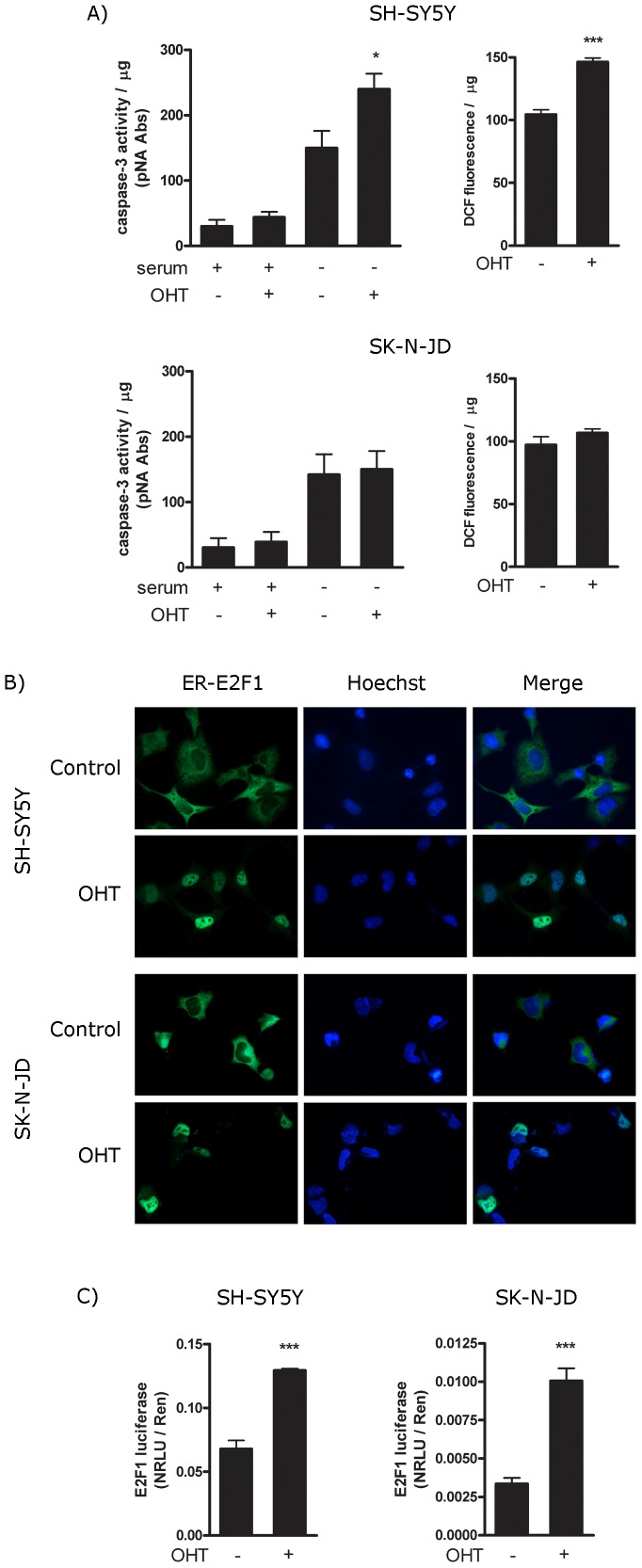
Apoptotic function of E2F1 is correlated with its ability to induce ROS accumulation. (A) Indicated SH-SY5Y and SK-N-JD neuroblastoma ER-E2F1 stable cell lines were treated (+) or not treated (−) with OHT, in the presence (+) or absence (−) of serum for 4 hours. Cell extracts were collected and lysed, and caspase-3 activities (left panels) and ROS levels (right panels) were analysed as described in Material and Methods. (B) Indicated SH-SY5Y and SK-N-JD neuroblastoma ER-E2F1 stable cell lines were treated (OHT) or not treated (control) with OHT for 4 hours. Cells were immunostained for E2F1 protein (red) and DAPI (blue). (C) Indicated SH-SY5Y and SK-N-JD neuroblastoma ER-E2F1 stable cell lines were transfected for 16 hours with a E2F luciferase reported construct and treated with (+) or without (−) OHT. Luciferase was measured and normalized for *Renilla* luciferase readings in the same extracts and the values were indicated. Results are presented as Mean ± SEM, for n = 4. In all figures, Student’s *t*-test values of *p<0,05, **p<0,01 and ***p<0,0001 were considered statistically significant.

### RT^2^ Profiler PCR Array

RNA from SH-SY5Y and SK-N-JD ER-E2F1 stable cell lines, treated in the different conditions, was extracted using the RNeasy® Mini Kit (SABiosciences, Frederick, MD) and converted to cDNA by using the RT^2^ First Strand Kit (SABiosciences, Frederick, MD). Quality of cDNA was confirmed with the RT^2^ Profiler PCR Array own controls, which tests for RNA integrity, inhibitors of reverse transcription and PCR amplification and genomic and general DNA contamination. Gene expression was analyzed in every cell condition by using the Human Oxidative Stress and Antioxidant Defense RT^2^ Profiler PCR Array (SABiosciences), which profiles the expression of 84 genes related to oxidative stress. PCR amplification was performed on an ABI Prism 7900HT sequence.

**Figure 7:**
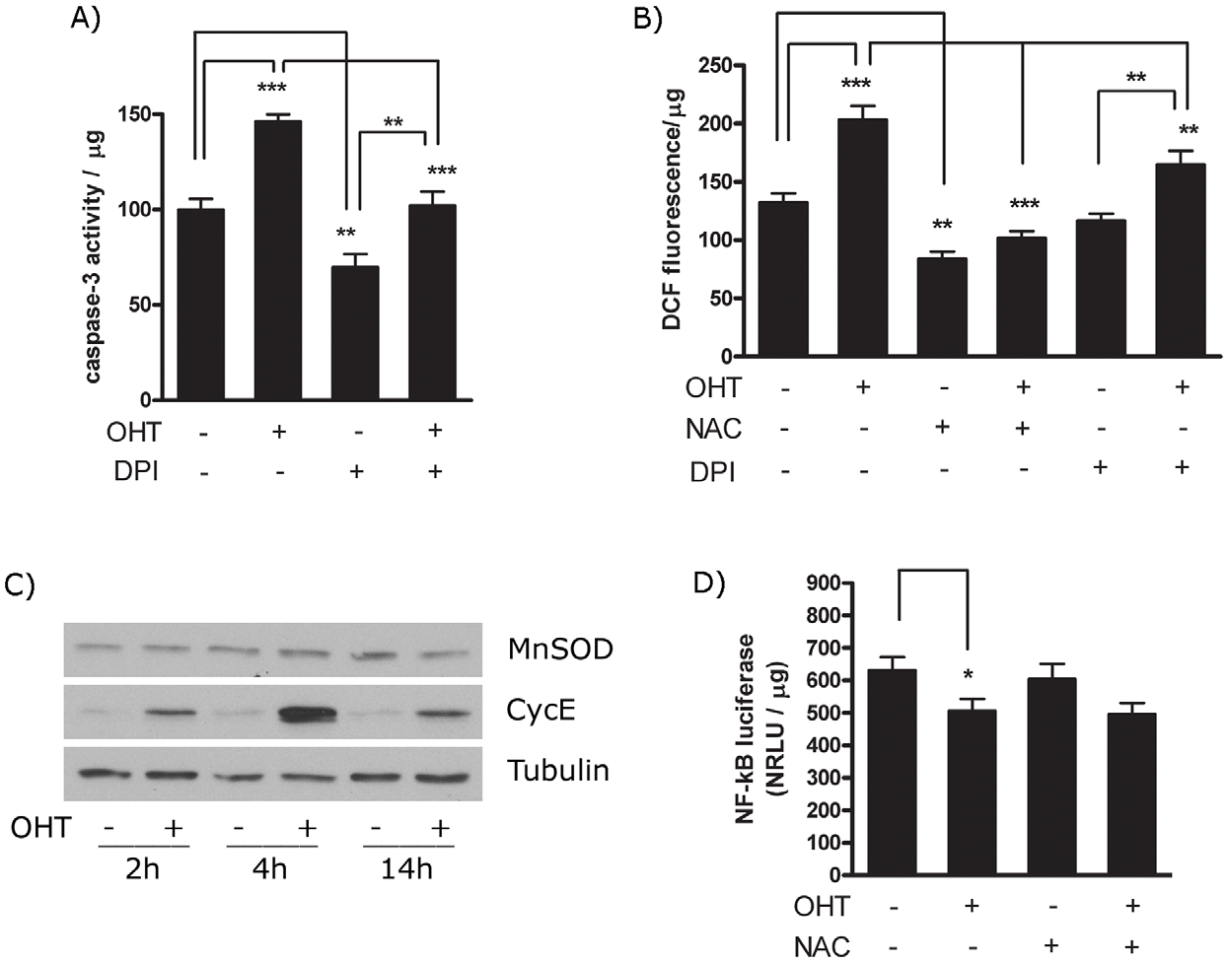
E2F1 does not regulate the ROS levels through Nox, but is capable to modulate NF-κB activity. (A) PC12 ER-E2F1 cells were serum deprived and treated with (+) or without (−) OHT in the presence (+) or absence (−) of DPI for 4 hours. After cell collection and lysis, caspase-3 activity of cell extracts was analysed by using the *p*NA colorimetric assay as indicated in Material and Methods. (B) Stable ER-E2F1 PC12 cells were treated with (+) or without OHT, in the presence (+) or in the absence (−) of 1 mM NAC, or DPI for 4 hours. ROS levels were analysed by using the oxidation-sensitive fluorescent probe H2DCFDA as described in Material and Methods. (C) Serum-deprived cells were treated (+) or not treated (−) with OHT for the indicated hours. Expression of the referred proteins was determined by Western Blot analysis. (D) Stable ER-E2F1 cells were transfected for 16 hours with a NF-κB reported construct and treated with (+) or without (−) OHT in the presence (+) or absence (−) of 1 mM NAC for 4 hours. Luciferase was measured and normalized with protein content. Results are presented as Mean ± SEM, for n = 3. In all of the figures, data are compared as indicated individually. Student’s *t*-test values of *p<0,05, **p<0,01 and ***p<0,0001 were considered statistically significant.

### Transfection and Luciferase Assay

Cells were transfected with 4 µg of YFP-Bax plasmid (kindly provided by Dr. Andrew Gilmore), or ER-E2F1 plasmid (kindly provided by Dr. Kristian Helin) or pcDNA.3-3HA-hBclxL and pEIGW-SK-mFLIP-FLAG plasmids (kindly provided by Dr. Joan Comella), or NF-κB and E2F luciferase reporter vectors using 10 µl of Lipofectamine 2000 (Invitrogen, USA) in accordance with the manufacturer’s instruction. To obtain the ER-E2F stable cell lines, cells were re-suspended and incubated in D-MEM selective medium, containing 10% serum and 750 µg/ml Geneticin Selective Antibiotic (G418) (Gibco, UK). After 15 days, the transfected stable cell line was obtained and maintained in the D-MEM selective medium. Luciferase activity was measured using either the Luciferase Assay kit (Promega, USA) in accordance with the manufacturer’s recommendations and normalized with protein content in the case of the NF-κB-promoter assay, or the renilla luciferase in the case of the E2F1-promoter assays.

**Figure 8:**
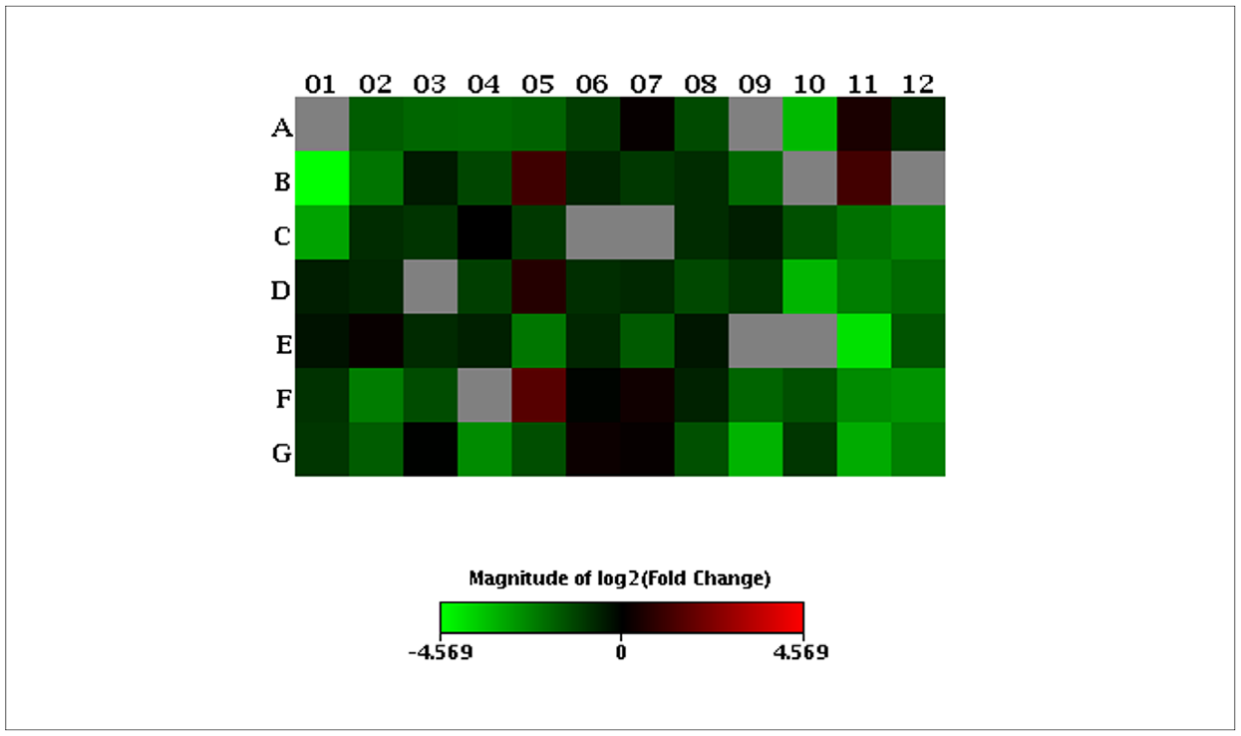
Heatmap of oxidative and antioxidative genes regulated by E2F1. mRNA from ER-E2F1 SH-SY5Y was isolated from OHT treated or not treated cells and quantified using the RT^2^ qPCR array platform as described in Material and Methods. All OHT regulated genes were compared against controls, using Hypoxanthine phosphoribosyltransferase 1 gene as the reference. The relative magnitude of expression is indicated on a spectrum ranging from minimum (green) to the maximum detected (red). All genes tested in the array are listed on Table S1.

### Localization of YFP-Bax

Cells transfected with YFP-Bax plasmid vector were carefully resuspended in culture medium and redistributed into 6-well plates covered with glass coverslips and coated with poly-DL-ornithine. Following cell adhesion, the cells were treated in the different conditions for 3 h, and during the last 15 minutes of incubation, the cells were stained for mitochondria with 50 nM of MitoTracker CMX-ROS (Invitrogen, USA). Finally, cells were fixed with 4% paraformaldehyde in PBS for 15 min at room temperature, washed with PBS, mounted with Fluoroshield (Sigma-Aldrich, USA) and visualized in a Leica DMI 4000 B microscope with the appropriate filters.

**Figure 9:**
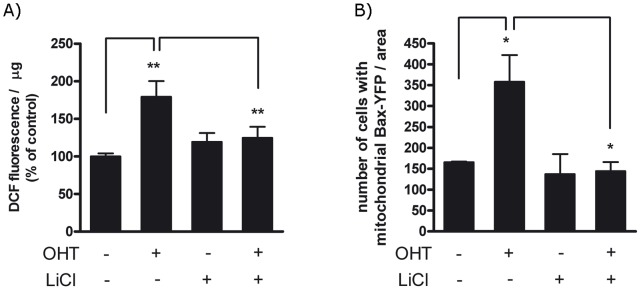
LiCl inhibits ROS production and Bax activation induced by E2F1. (A) PC12 ER-E2F1 cells were serum deprived and treated with OHT (+) or not (−) with OHT, in the presence (+) or in the absence (−) of 40 mM LiCl for 4 hours. ROS levels were analysed by using the oxidation-sensitive fluorescent probe H2DCFDA as described in Material and Methods. Results are presented as Mean ± SEM, for n = 4. (B) PC12 ER-E2F1 cells were transiently transfected with an YFP-Bax plasmid, serum deprived and treated with OHT (+) or not (−) with OHT, in the presence or absence of 40 mM LiCl for 3 hours. Quantification of the punctuated mitochondrial clusters of YFP-Bax achieved in the immunofluorescence assay is described in Material and Methods. Results are presented as Mean ± SEM, for n = 3. In all of the figures, data are compared as indicated individually. Student’s *t*-test values of *p<0,05 and **p<0,01were considered statistically significant.

### Immunofluorescence of Activated Bax

Cells were grown on poly-DL-ornithine-coated 6-well plates with glass coverslips and treated with OHT for 3 h. During the last 15 minutes of incubation, cells were stained for mitochondria with MitoTracker CMX-ROS at 50 nM (Invitrogen, USA). At the end of treatment cells were fixed with 4% paraformaldehyde during 15 min with constant agitation, washed with PBS, permeabilized for 2 min with 0,2% CHAPS and then incubated for 1 h with 1∶200 anti-Bax antibody 6A7 clone (BD Bioscience, USA) in 10% horse serum to detect mitochondrial activated Bax. After several washes in PBS, cells were incubated with the secondary antibody Alexa 488-conjugated anti-mouse IgG at 1∶100 dilution (Molecular Probes, USA) in 10% horse serum for 1 h, washed again, and then mounted with Fluoroshield (Sigma-Aldrich, USA). The fluorescence was detected in a Leica DMI 4000 B microscope with the appropriate filters.

### Mitochondria Isolation and Crosslink of Bax

Cells were washed once in PBS, suspended in 500 µl hypotonic buffer (10 mM NaCl, 1,5 mM MgCl2, 10 mM Tris-HCl, pH 7,5) and incubated on ice for 10 min. Cells were ruptured with 20 strokes with a tight-fitting pestle in a Dounce homogenizer, 400 µl of 2,5x buffer B (525 mM mannitol, 175 mM sucrose, 12,5 mM Tris-HCl, pH7,5, 2,5 mM EDTA, ph 7,5) was added, and mitochondria were isolated by differential centrifugation. Samples were centrifugated at 1,300×g for 10 min at 4°C, supernatants were transferred to clean tubes, and centrifugation was repeated twice. The resulting supernatants were then centrifugated at 17,000×g for 20 min at 4°C to pellet mitochondria. The mitochondrial pellet was washed once with 1x buffer B and re-centrifugated. Cross-linking reactions were then performed on the mitochondrial pellets suspended in 200 µl PBS by treatment with 1 mM bismaleimidohexane (Pierce, USA) for 30 min at room temperature. Mitochondria were pelleted and suspended in sample buffer supplemented with 10 mM DTT to quench the cross-linking reaction. Samples were then subjected to immunoblot analysis with anti-Bax antibody (Santa Cruz Biotechnology, USA) to detect oligomeric Bax.

### Detection of ROS

Cells were incubated with 2,5 µM H2DCFDA (Invitrogen, USA) at 37°C in HBSS without red phenol, lysed for 10 min at 4°C with lysis buffer (25 mM HEPES pH 7,5, 60 mM NaCl, 1,5 mM MgCl2 and 0,1% Triton X-100) and then transferred in duplicate to a 96-well plate. Fluorescence of the DCF subproduct was measured in a Microplate Fluorescence Reader Fluorstar Optima and expressed as percentage of control after correction with protein content.

## Results

### Bax Activation is Implicated in the E2F1-induced Apoptosis

Previously we demonstrated that in PC12 cells over-expression of E2F1, employing the ER-E2F1 fusion protein, induces apoptosis through activation of caspase-3 [16]. To investigate the mechanism by which E2F1 controls apoptosis, we asked whether this was through an extrinsic or intrinsic pathway, both of which have been shown to be induced by E2F1, depending on cell type [11]–[13], [17]. The ER-E2F1 fusion protein is expressed in the cytosol; with 4-hydroxytamoxifen (OHT) treatment inducing its translocation to the nucleus [16]. Induction of the extrinsic pathway was measured by following the activation of caspase-8. As is shown in Fig. 1A caspase-8 activity did not change upon treatment with OHT either in the presence or absence of serum. In agreement with previous reports that serum deprivation induces the activation of the extrinsic apoptotic pathway, increased caspase-8 activity was found in all of conditions where serum was absent [18]. Caspase-8 activity was also determined by measuring the endogenous cleavage of Bid, a well-known substrate of caspase-8 [19]. As is shown in Fig 1B, serum deprivation induced the appearance of the proteolytic form of Bid, tBid, to mitochondria, however, treatment with OHT did not produce any further increase. In addition, we measured the expression of Bid on the total cell extract after serum starvation and OHT addition. As shown in Fig S1, in these conditions we were not able to detect the cleavage of Bid and Bid levels remained constant. Probably, only a small amount of Bid is cleavage and translocate to mitochondria compared to the full Bid protein and as a consequence, the change on full Bid proteins are difficult to detect. That OHT treatment led to E2F-transcriptional activation is demonstrated by the induction of of Cyc E, an E2F1 cell cycle target [20]. In parallel, addition of OHT led to increased caspase-3 activity as shown in Fig 1C and to the induction of apoptosis as measured by the appearance of sub-diploid DNA peak by Scatter analysis as shown in Fig. S2. These findings argue that E2F-induced apoptosis is mediated by an intrinsic, rather than an extrinsic pathway. In order to confirm the involvement of the intrinsic pathways on the E2F1-induced apoptosis, we measured the effect of over-expression of Bcl-xL on this process. As is shown in Fig 2A, over-expression of Bcl-xL arrested OHT-induced apoptosis. By contrast, over-expression of c-FLIP, a caspase homologue that is thought to be an inhibitor of death-receptor-mediated apoptosis, did not arrest OHT-induced apoptosis as shown in Fig. 2B.

**Table 1:** mRNA levels changes after OHT addition in SH-SY5Y cells.

*Unigen*	*Symbol*	*Description*	*Fold Change*			
			*Without LiCl*		*With LiCl*	
			*OHT #1*	*OHT #2*	*OHT #1*	*OHT #2*
*Genes downregulated*						
Hs.95120	*CYGB*	Cytoglobin	0.13	0.01	0.74	1.34
Hs.502823	*PRDX5*	Peroxiredoxin 5	0.21	0.02	3.03	6.08
Hs.502917	*CCS*	Copper chaperone for superoxide dismutase	0.05	0.19	6.10	10.87
Hs.728817	*TXNRD1*	Thioredoxin reductase 1	0.18	0.08	3.63	4.90
Hs.134602	*TTN*	Titin	0.22	0.05	3.63	6.11
Hs.443430	*TXNRD2*	Thioredoxin reductase 2	0.19	0.21	2.38	7.36
Hs.251386	*PRG3*	Proteoglycan 3	0.23	0.20	5.40	3.77
Hs.234742	*LPO*	Lactoperoxidase	0.32	0.12	3.44	8.92
Hs.631770	*DGKK*	Diacylglycerol kinase, kappa	0.17	0.33	0.64	0.94
Hs.239	*FOXM1*	Forkhead box M1	0.31	0.25	0.70	1.04
Hs.146559	*ANGPTL7*	Angiopoietin-like 7	0.19	0.41	0.65	0.95
Hs.654439	*APOE*	Apolipoprotein E	0.25	0.36	0.69	1.64
Hs.128856	*SCARA3*	Scavenger receptor class A, member 3	0.43	0.20	1.14	2.32
Hs.180909	*PRDX1*	Peroxiredoxin 1	0.25	0.43	0.79	1.46
Hs.467554	*TPO*	Thyroid peroxidase	0.33	0.43	2.32	2.60
*Genes upregulated*						
Hs.333358	*GPR156*	G protein-coupled receptor 156	3.94	1.31	0.83	1.22

RT^2^Profiler human oxidative stress and antioxidant defense PCR Arrays (Bioscience) were performed according to the manufacture’s protocols. Expression levels were compared between with and without OHT addition. Hypoxanthine phosphoribosyltransferase 1 gene was used as control for each gene expression calculation, and the extent of change in the expression of each gene was calculated by the ΔC_t_ method. Only genes whose expression was downregulated at least 2 fold are shown in the Table. We have also removed all genes whose expression change significantly between duplicates. When ΔC_t_ was over 12 and therefore expression was thought to be extremely low, the gene was omitted from analysis.

The intrinsic apoptotic pathway is dependent on the balance between pro- and anti-apoptotic members of the Bcl-2 superfamily members. Previous studies demonstrated that over-expression of E2F1 regulates the expression of many pro- and anti-apoptotic proteins [21]. Because the transcriptional regulation of these genes and their contribution to E2F1-induced apoptosis is cell type dependent, we assessed the pattern of Bcl-2 expression after the E2F1-induction. Cells were serum deprived and the expression of Bcl-2 target genes was determined at different times after OHT addition. CycE expression was used as a positive control of E2F1-transcriptional activity regulation. As it is shown in Fig. 3A, induction of E2F1 led to the expression of BimL, but none of the other members of the Bcl-2 family including Bax, Bcl-xL, Noxa, Puma, and Mcl1. RT-PCR analysis demonstrated that the increase in BimL protein expression correlated with an increase in the expression of its cognate mRNA as shown in Fig. 3B. The levels of p53, N-Myc and GSK3β proteins, whose expressions are regulated by E2F1 in other experimental systems, did not change under our experimental conditions [2], [22], [23]. These data demonstrate that E2F1 regulates BimL expression by regulating its mRNA levels, and suggest a role of this protein in the apoptotic function of E2F1 in PC12 cells.

Re-localization of Bax from the cytosol to the mitochondria represents one of the first steps in programmed cell death in neurons [24]. To investigate whether over-expression of E2F1 promotes the translocation of Bax to mitochondrial compartment, cytosolic and mitochondrial extracts were obtained at different times after OHT treatment and the amount of mitochondrial-associated Bax was determined. As shown in Fig. 3C, addition of OHT induced the re-localization of Bax to mitochondria in a time-dependent manner, which was paralleled by a decrease in the amount of anti-apoptotic Bcl-2 member, Bcl-xL, localized with mitochondria. Hydrogen peroxide, a potent inducer of apoptosis, was used as a positive control for Bax and Bcl-xL translocation, and Cox IV, as a protein loading control. To confirm the role of E2F1 on Bax intracellular distribution, we evaluated its localization by transfecting PC12 ER-E2F1 stable cells with a YFP-Bax fusion construct. As is shown in Fig 4A and B, most of the untreated cells displayed a cytosolic pattern of fluorescence. However, the addition of OHT produced significant changes in the distribution of the fluorescence: from a diffuse, cytosolic distribution to a punctuated pattern of fluorescence. This change was shown to represent the redistribution of Bax to the mitochondria, as demonstrated by the overlap between YFP-Bax fluorescence and the localization of mitochondria revealed by staining with MitoTracker.

Insertion of Bax into the mitochondria implies changes in Bax conformation, together with its oligomerization; processes which are essential for its apoptotic function [24]–[26]. To examine whether E2F1 induced a change in the conformation of Bax, we carried out an immunofluorescence labeling analysis by staining untreated and OHT treated cells with the conformation-sensitive Bax 6A7 antibody. This antibody recognizes the NH2-terminal Bax epitope that is exposed to the external mitochondria membrane as a consequence of the Bax conformational change [26]. As shown in Fig. 4C, 6A7 immunofluorescence label was absent in untreated cells, however it was present in OHT-treated cells. The 6A7 immunofluorescence label localized with the subcellular marker MitoTracker, demonstrating that active Bax resides on mitochondrial vesicles. Furthermore, analysis of Bax oligomers by chemical crosslinking, revealed that the addition of OHT induced the appearance of one band that possesses molecular weight coincident with the dimeric form of Bax (Fig. 4E). Thus, E2F1 activation induces mitochondrial translocation and activation of Bax, a response which parallels E2F1-mediated apoptosis. To determine the functional relevance of Bax in E2F1-apoptosis, we employed a commercially available peptide that was previously shown to bind directly to Bax, prevent its mitochondrial translocation, and inhibit Bax mediated apoptosis [27]. As shown in Fig. 4F, treatment with the Bax-inhibitory peptide reduced OHT-induced apoptosis. These results point out an essential role of Bax on the apoptotic process driven by E2F1.

### Generation of Reactive Oxygen Species is Essential for E2F1 Induced Apoptosis

Reactive oxygen species (ROS) have been implicated in the regulation of neuronal apoptosis in several neurodegenerative diseases. To examine the role of ROS in E2F1-dependent apoptotic process [28], its accumulation was measured fluorometrically using the oxidation-sensitive fluorescent probe 2′-7′-dichlorodihydrofluorescein diacetate (H_2_DCFDA) [26]. H_2_DCFDA is hydrolyzed and oxidized to fluorescent 2′,7′-dichlorofluorescein (DCF) by ROS. Treatment of PC12-ER-E2F1 cells with OHT caused an increase in ROS as evidenced by the dramatic increase of DCF fluorescence (Fig 5A). Levels of DCF were increased in a time-dependent manner, indicating an accumulation of ROS during the apoptotic process. To answer the question as to whether ROS is involved in the induction of apoptosis by E2F1, we determined the effect of *N*-acetylcysteine (NAC), a well-known free radical scavenger, on this process. Apoptosis was determined by measuring the change on caspase-3 activity as reflected by DEVD-AFC cleavage. As shown in Fig 5B, the addition of NAC prevented OHT induced-activation of caspase-3. As predicted from our earlier studies, the addition of either LiCl or serum to the cell medium inhibited the E2F-induced apoptosis [16]. To determine whether ROS is responsible for Bax activation, we measured the effect of NAC on the translocation of transfected Bax-YFP fusion construct into mitochondria. Results demonstrate that such treatment decreased the re-localization of Bax from the cytosol to the mitochondria, as measured by the YFP-Bax fluorescence assay (Fig. 5C). We excluded any inhibitory role of NAC on the transcription activity of E2F1 by confirming that the transcription levels of E2F reported construct B-Myb-luciferase did not change after NAC treatment (results not shown). These results strongly imply that production of ROS is essential for the apoptotic effect of E2F1 in PC12 cells and suggest that generation of ROS could be the initiation event for the apoptotic response.

Next we investigated whether this finding could be extended to other neuron cell lines. To this end, we stable transfected SH-SY5Y and SK-N-JD neuroblastoma cells with ER-E2F1 expression vector and measured apoptosis and ROS production after the addition of OHT. As shown in Fig. 6A, the presence of OHT increased caspases-3 activity as well as ROS production in SH-SY5Y cells. In contrast in SK-N-JD cells, activation of E2F1 by the addition of OHT did not affect ROS production, and interestingly, there was no effect on apoptosis. This was not due to a failure to induce E2F1, as immunofluorescence studies show that the addition of OHT induced the translocation of ER-E2F1 to the nuclei equally in both cell types (Fig. 6B). Likewise, co-transfection of both cell types with ER-E2F1 and an E2F-luciferase reporter shows that OHT induced E2F1 transcriptional activity to a similar extent (Fig. 6C). These data demonstrate that the differential ability of E2F1 to induce apoptosis in SH-SY5Y versus SK-N-JD cells correlates with its ability to generate ROS.

### E2F1 Regulates the Expression of a Large Number of Genes Involved on ROS Metabolism

The steady-state levels of ROS are controlled by the rate of ROS production and clearance by scavenging mechanisms. The major ROS-generating systems in cells include the mitochondria and the seven known NOX isoenzymes [29]–[31]. Although ROS is primarily generated following release of electrons from the electron transport chain at the mitochondria, NOXs are capable of generating ROS in a highly regulated manner [29]–[31]. In order to investigate whether the intracellular accumulation of ROS after induction of E2F1 is due to the upregulation of NOX, we have studied the effect of NOX-inhibitor diphenyleneiodonium (DPI) on the E2F1-induced apoptosis by measuring caspase-3 activation [32]. As shown in Fig 7A, DPI treatment repressed caspase-3 activity in the absence or presence of OHT, however there were significant differences in the extent of caspase-3 repression. Similarly, DPI treatment only led to partial diminishing of ROS levels in the presence of OHT as shown in Fig 7B. The inability of DPI to impair ROS generation, suggests that E2F1 regulates ROS production by mechanisms distinct from the NOXs.

As previous reports demonstrated that E2F1 represses the expression of antioxidative enzyme Mn superoxide dismutase (MnSOD) through inhibition of NF-κB [14], we next evaluated whether regulation of MnSOD was implicated in the E2F1-induced apoptotic response. To test this possibility, the expression of MnSOD and mRNA levels were determined at different times following OHT treatment, with CycE mRNA expression used as a positive control. As shown in Fig 7C, the levels of MnSOD protein remained constant after OHT induction and no change in MnSOD mRNA levels were detected (data non shown). However, it is possible that other antioxidative enzymes, which are transcriptional targets of NF-κB, were responsible for the effects of E2F1. To test if E2F1 modulates NF-κB activity, and, as a consequence ROS content, we transfected ER-E2F1 PC12 cells with a NF-κB luciferase reporter construct. The results show that the addition of OHT induced a significant reduction of NF-κB activity as shown in Fig. 7D. These results exclude the involvement of MnSOD on the accumulation of ROS by E2F1, and suggest a potential role for NF-κB in this process.

Because E2F1 regulates the transcription of extensive number of genes, to fully understand the molecular mechanism mediating the production of ROS by E2F1, we compared the mRNA profiling of genes involved in ROS metabolism between control cells and those exposed to OHT. To this end, mRNA levels of 84 genes implicated in ROS production were analyzed in SH-SY5Y cells by the Human Oxidative Stress and Antioxidant Defense TR^2^ PCR array (SABioscences). This cell line was chosen because its human origin allows us to use a commercial PCR array. Upon OHT treatment, these gene expression profiles were significantly altered, suggesting that both oxidative and antioxidant genes are targets of E2F1. A heat map is shown in Fig. 8 displaying a visual representation of these changes on gene expression. Of these genes, fifteen display significant down-regulated response to OHT (more than two-fold difference in expression and only two were found up-regulated as shown in Table 1). The transcriptional profile reveals that antioxidant functions are down-regulated, suggesting that this global change could be responsible for the increase of ROS levels. The same expression analysis was carried out in SK-N-JD neuroblastoma cells, which have a very low apoptotic response to activation of E2F1. Upon OHT treatment, in contrast to SH-SY5Y cells, the profile of 84 genes was not significantly affected as shown in Table S2. This suggests that oxidative and antioxidative genes of both cells respond differently to E2F1 and may explain their different apoptotic behavior.

### GSK3β Activity is Essential for the ROS Production Induced by E2F1

As we previously demonstrated that GSK3β is required for apoptotic function of E2F1 [16], we were interested to assess whether it was required to modulate ROS levels induced by E2F1. As shown in Fig 9A, inhibition of GSK3β activity by LiCl blocked the production of DCF induced by E2F1, suggesting that GSK3β is also necessary for ROS production. Consistent with this finding, the presence of LiCl prevented the translocation of Bax-YFP to the mitochondria after E2F1 activation as shown in Fig. 9B. In Fig. S3 we show similar results using CT99021, another GSK3β inhibitor. In order to understand the inhibitory effect of LiCl on the induction of ROS by E2F1, we determined change in mRNA profiling after OHT treatment in the presence of LiCl. As is shown in Table 1, upon treatment with LiCl, the negative effect of E2F1 on the expression level of the previously mentioned antioxidative genes was abolished. These findings argue that the effects of E2F1 on ROS are mediated by GSK3β.

## Discussion

Although E2F1 is over-expressed in most neuronal tumors, studies from many laboratories, including ours, showed that it is also an important mediator of neuronal apoptosis [16]. In order to understand this seemingly counterintuitive role, we investigated the mechanism by which E2F1 promotes apoptosis in neuronal tumor cells. In this study we show that apoptotic function of E2F1 is mediated through an intrinsic signaling pathway, in which the translocation of Bax to mitochondria is the key event. We also provide evidence against the participation of an extrinsic mechanism in mediating the effects of E2F1. In this respect, others have reported that E2F1 is able to modulate the apoptotic response by either FAS ligand or tumor necrosis factor [10]–[12]. Our results show that activation of E2F1 did not alter the activity of caspase-8, strongly suggesting that death receptors are not involved in the E2F1 apoptotic activity in PC12 cells. Consistent with this finding, E2F1 was not able to induce changes in the levels of Bid, a BH3-only protein whose cleavage is triggered by caspase-8. Moreover, over-expression of Bcl-xL arrested E2F1-induced apoptosis; and, by contrast, over-expression of c-FLIP, did not has any effect on OHT-induced apoptosis. These results demonstrate the implication of intrinsic pathway on the apoptosis induced by E2F1 and point out an essential role of mitochondria in this process.

Members of the Bcl-2 family play an important role in regulating mitochondrial integrity. The involvement of intrinsic pathway in the apoptosis response induced by E2F1 is largely based on the fact that several Bcl2 family members have been reported to be directly or indirectly regulated by E2F1 [13]. Here we show that E2F1 induced the translocation of the pro-apoptotic protein Bax to the mitochondria, without affecting total Bax levels. These results are consistent with previous data demonstrating that, in response to distinct forms of cell death stimuli, Bax is redistributed from cytosol to the mitochondria where it induces a decline in the mitochondria membrane potential followed by cytochrome c release and caspase 3 activation [33]. The capacity of Bax to promote neuronal cell death has been reported in multiple neuronal populations, however the mechanism by which Bax alters mitochondria membrane potential is not well defined. It is now accepted that, after mitochondria translocation, Bax protein forms oligomers that permeabilize the mitochondria membrane [24]–[26]. Here we show that E2F1 is also able to induce the oligomerization of Bax, and we were able to detect the conformation change associated with its insertion into the mitochondria. Inhibition of Bax activity by treatment with the Bax-inhibitory peptide reduced E2F1-induced apoptosis and point out an essential role of Bax in this process.

Pro-apoptotic genes such as PUMA, Noxa, Bim, HrK, and Bad have been reported to be up-regulated by E2F1 induction, in contrast to the anti-apoptotic member Mcl-1, which is repressed. The list of Bcl-2 targets is still growing, and the functional roles of the individual members are dependent on cell type [21]. BimL was found to be the only Bcl-2 member whose expression levels changed after E2F1 induction in PC12 cells. Our results are consistent with previous reports demonstrating that E2F activity controls the transcription of BimL by regulating the levels of myb, a transcription factor that controls BimL transcription [34]. BimL is a pro-apoptotic BH3-only member of Bcl-2 family that is required for initiation of neuronal apoptosis induced by NGF withdrawal or by other specific stimuli including UV and ER stress [35]–[37]. The mechanism by which BimL activates apoptosis is still unclear. However, several reports have noted that BimL activates apoptosis through Bax and it has been suggested that high levels of BimL displace Bcl-xL in the mitochondria, promoting the insertion of Bax into the mitochondrial membrane [38]–[39]. Interestingly, we found that E2F1 induction diminished the mitochondrial enrichment of Bcl-xL. It is possible that the increase of BimL content, induced by E2F1, facilitates the re-localization of Bcl-xL. In agreement with this model, activation of Bax by E2F1 could, in part, induce the re-distribution of the Bcl-2 members through increasing of BimL levels.

In this study we demonstrated that the apoptotic action of E2F1 depends on the accumulation of ROS. Numerous reports have linked oxidative stress and ROS with neuronal apoptosis, in most cases Bax plays a central role [31]. Activation of Bax, by apoptotic stimuli can induce an increase in the production of O_2_, by blocking the electron transport chain, the main physiological source of intracellular ROS [39]. Although we do not exclude the participation of Bax in ROS generation, our results suggest that production of ROS by E2F1 occurs by a Bax-independent mechanism. We demonstrated that diminishment of ROS levels by NAC repressed not only E2F1-induced apoptosis, but also the translocation of Bax to the mitochondria, implying that ROS acts upstream from Bax activation. The molecular mechanism by which ROS leads to Bax activation is unknown. In colon adenocarcinoma cells, it has been reported that H_2_O_2_ induces Bax activation through modulating the oxidative state of Bax cysteine 62 [40]. It is also possible, as described in other apoptotic settings, that modification of intracellular pH, produced by an increase in ROS levels, induces Bax translocation from the cytosol to mitochondria [41]. Moreover, ROS accumulation can induce the activation of specific signal transduction pathways, such as those mediated by Jun NH_2_- terminal kinase or p38 MAP kinases, and as a result, phosphorylate and activate Bax or other Bcl-2 members that are involved in Bax activation [42], [43].

We also attempted to identify the possible sources of increased ROS concentration by Real Time PCR array of genes involved in ROS metabolism and oxidative stress. We found that many of the genes responsible for scavenging ROS were down-regulated after E2F1 induction in SH-SY5Y cells. These genes included several peroxiredoxins, thioredoxin reductase 1, cooper chaperone for superoxide dismutase and peroxidases, such a lactoperoxidase and cytoglobin. Cytoglobin, whose mRNA levels changed the most dramatically, is responsible for intracellular oxygen storage as well as the transfer and sensing of O_2_ [44]. Peroxiredoxins play an important defensive role in degenerative brain diseases and neuronal cell death in adults [45], [46]. Copper chaperones for superoxide dismutase activates the ROS-scavenging function of SOD1 by delivering of Cu^2+^ and has been proposed for treatment of Alzheimer’s disease [47]. Thioredoxin reductase 1 is a major antioxidant and redox regulator in mammalian cells and has an essential role in mammalian development and cancer [48]. Interestingly, most of the down-regulated genes also play an antiapoptotic role; this is the case for cytoglobin, peroxiredoxin 1, 5, the copper chaperone for superoxide dismutase, and thioredoxin reductase [48]–[52]. The diminished expression of these genes after E2F1 activation could facilitate the loss of the protective role against cell death. To our knowledge, none of these genes have been reported previously to be regulated by E2F1. However, some contain evident E2F binding sites in their promoter region, such as cytoglobin [53]. Moreover, superoxide dismutases, peroxidases, peroxiredoxin are targeted by NF-κB signaling pathway. A previous report demonstrated that through inhibition of NF-κB activity, E2F1 represses the expression of the superoxide dismutases, MnSOD, [14]. We did not observe any change in the expression of MnSOD, following E2F1 induction despite the fact that E2F1 inhibited NF-κB activity under the conditions we employed. These data strongly support a model in which the apoptotic function of E2F1 is regulated by the NF-κB pathway.

The individual role of each of these genes on the production of ROS remains unknown but probably the oxidative response to E2F1 is due to the sum of their action. Although the majority of the genes responsible for ROS scavenging were down-regulated, others such as dual oxidase 2 and the peroxidase G protein-couple Receptor 156 were up-regulated. An imbalance between the differential expressions of these genes can lead to the accumulation of ROS and drive, as a consequence, the activation of the apoptotic response. In this respect, the lack of ROS production and apoptosis induction observed in SK-N-JD cells after E2F1 activation could be due to the inability of E2F1 to down-regulated the expression of ROS scavenging genes.

Several growth factors critical to cell survival and proliferation are known to signal through ROS-dependent mechanisms involving NOX [17], [54]. One isoform of NOX, NOX4, is transcriptionally regulated by E2F in vascular smooth muscle cells [15]. We did not detect any change on NOX4 mRNA and protein expression following E2F1 induction in PC12 cells (data not shown). Treatment of cells with DPI, a nonspecific inhibitor of all the Nox isoforms, but also of multiple electron transporters, was not able to reduce E2F1-induced ROS accumulation or apoptosis. This result is concordance, as mentioned above, with the suggestion that genes responsible for ROS scavenging are regulated by E2F1.

Previously we showed that apoptotic action of E2F1 required GSK3β [16]. Here we demonstrate that GSK3β activity is essential for the ROS production and Bax activation. Inhibition of GSK3β by LiCl also changed the pattern of expression of E2F1-regulated genes. Many of the genes responsible for scavenging ROS that were down-regulated after E2F1 induction in SH-SY5Y cells did not change, or were found at higher levels, when GSK3β activity was abolished. This change in the expression profile could explain the inhibitory effect of GSK3β on the ROS production and, as a consequence, the induction of apoptosis by E2F1. The results obtained are in agreement with previous studies showing that inhibitors of GSK3β activity or its depletion by siRNA treatment increase the transcriptional level of E2F1-regulated promoters. However, the fact that GSK3β can directly modulate transcription of E2F1-response genes, does not exclude the possibility that GSK3β could act downstream of E2F1, and regulate the activity of apoptotic proteins essential for cell fate decisions. Pro-apoptotic substrates for GSK3β have not been well characterized. Interestingly, Bax is directly phosphorylated by GSK3β; inhibition of GSK3β activity suppressed Bax mitochondrial translocation, conformational activation and cytocrome c release [55]. Moreover, inhibition of GSK3β can also lead to nuclear accumulation of beta-catenin and interfere with E2F1 responses, as it has been suggest in Myc/E2F1-driven hepatocarcinogenesis model [56].

The final decision of whether E2F1 induces apoptosis is a consequence of the integration of several cellular signals, which are cell type dependent. Over-expression of E2F1 in the neuroblastoma cell line SH-SY5Y induced apoptosis but not in neuroblastoma cell line, SK-N-JD, suggesting that the high activity of E2F1 itself does not cause apoptosis. This differential behavior correlates with the ability of E2F1 to generate ROS in SH-SY5Y, but not in SK-N-JD. From this result and all the other results we can conclude that generation of ROS is essential for the apoptotic function of E2F1. We think that this finding can lead to a better understanding of the seemingly contradictory role of E2F1 as an apoptotic agent as well as a cell cycle activator.

## Supporting Information

Figure S1 **Expression analysis of Bid after OHT treatment in ER-E2F1 PC12 cells.** Serum-deprived (−) or not (+) cells were treated (+) or not (−) with OHT for 8 hours. Expression of the indicated proteins was determined in total cell extract by Western Blot analysis.
(TIF)

Figure S2 **E2F1 induces the appearance of sub-diploid DNA peak.** Stable ER-E2F1 PC12 cells were treated or not treated with OHT in the presence or in the absence of serum. Analysis of cell cycle was achieved by forward light scatter and was quantified the subdiploid DNA peak of cells at the indicated conditions.
(TIF)

Figure S3 **CT99021 inhibits ROS production and Bax activation induced by E2F1.** (A) PC12 ER-E2F1 cells were serum-deprived and treated with (+) or without (−) OHT in the presence (+) or in the absence (−) of 10 µM CT99021 for 4 hours. ROS levels were analysed by using the oxidation-sensitive fluorescent probe H2DCFDA. Results are presented as Mean ± SEM, for n = 3. Student’s *t*-test value of ***p<0,0001 was considered statistically significant. (B) PC12 ER-E2F1 cells were serum-deprived and treated with (+) or without (−) OHT in the presence (+) or in the absence (−) of 10 µM CT99021 for 3 hours. Active Bax was detected by immunofluorescence using anti-Bax 6A7 clone antibody and anti-BAx 6A7 positives were quantified.
(TIF)

Table S1 **List of genes that are included on the Human Oxidative Stress and Antioxidant Defense RT^2^ Profiler PCR Array (Biosciences).**
(DOCX)

Table S2 **mRNA changes after OHT addition in SK-N-JD cells.-** RT^2^Profiler human oxidative stress and antioxidant defense PCR Arrays (Bioscience) were performed according to the manufacture’s protocols. Expression levels were compared between with and without OHT addition. Hypoxanthine phosphoribosyltransferase 1 gene was used as control for each gene expression calculation, and the extent of change in the expression of each gene was calculated by the ΔC_t_ method. We show the genes that were indicated on Table1.
(DOCX)
