# Supplementary figures and images for: ROS Production Is Essential for the Apoptotic Function of E2F1 in Pheochromocytoma and Neuroblastoma Cell Lines
Source: PLoS One. 2012 Dec 12;7(12):e51544. doi: 10.1371/journal.pone.0051544 (PMC3520901; doi:10.1371/journal.pone.0051544)

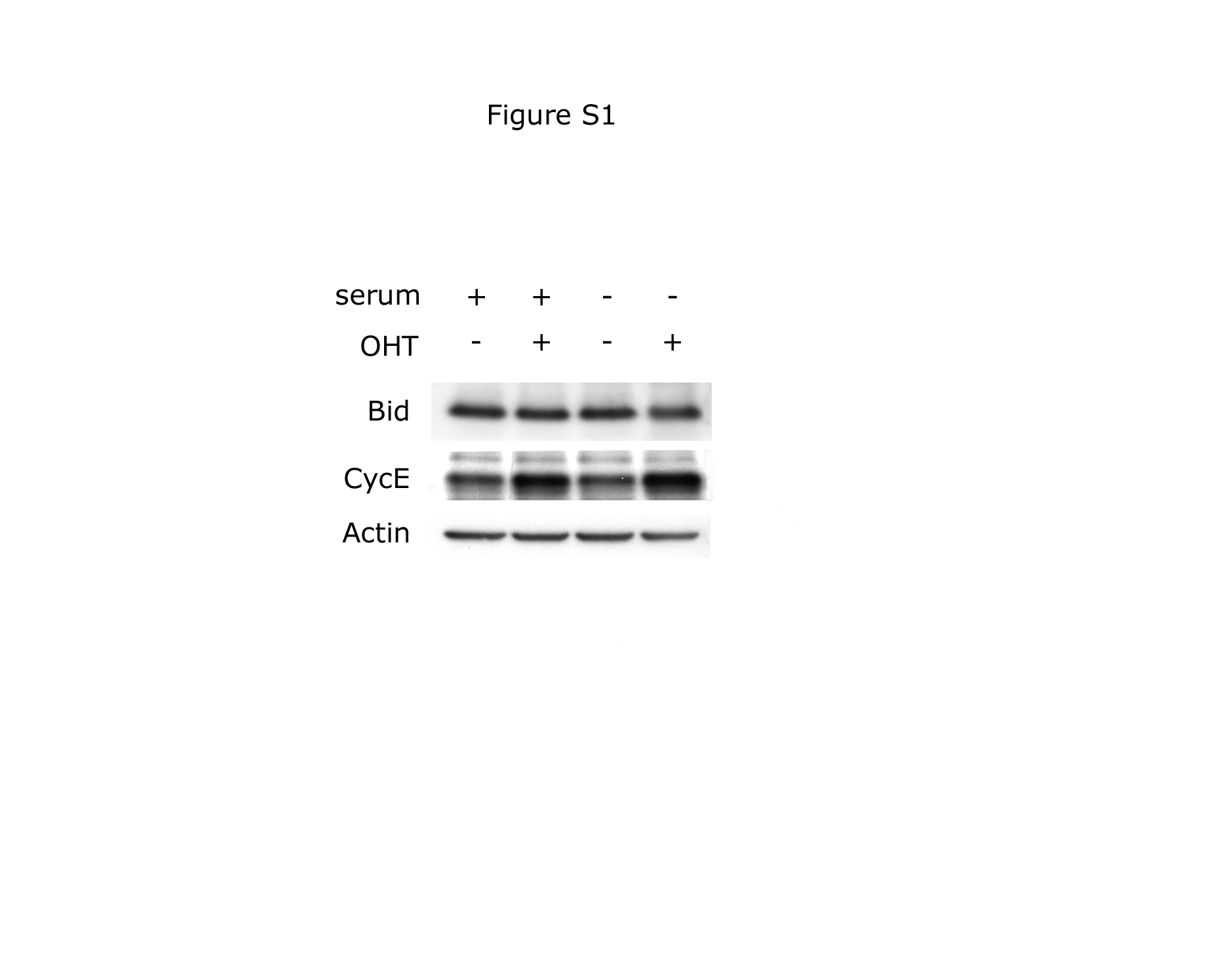

Supplement: Figure S1 — Expression analysis of Bid after OHT treatment in ER-E2F1 PC12 cells. Serum-deprived (−) or not (+) cells were treated (+) or not (−) with OHT for 8 hours. Expression of the indicated proteins was determined in total cell extract by Western Blot analysis. (TIF) [file pone.0051544.s001.tif]

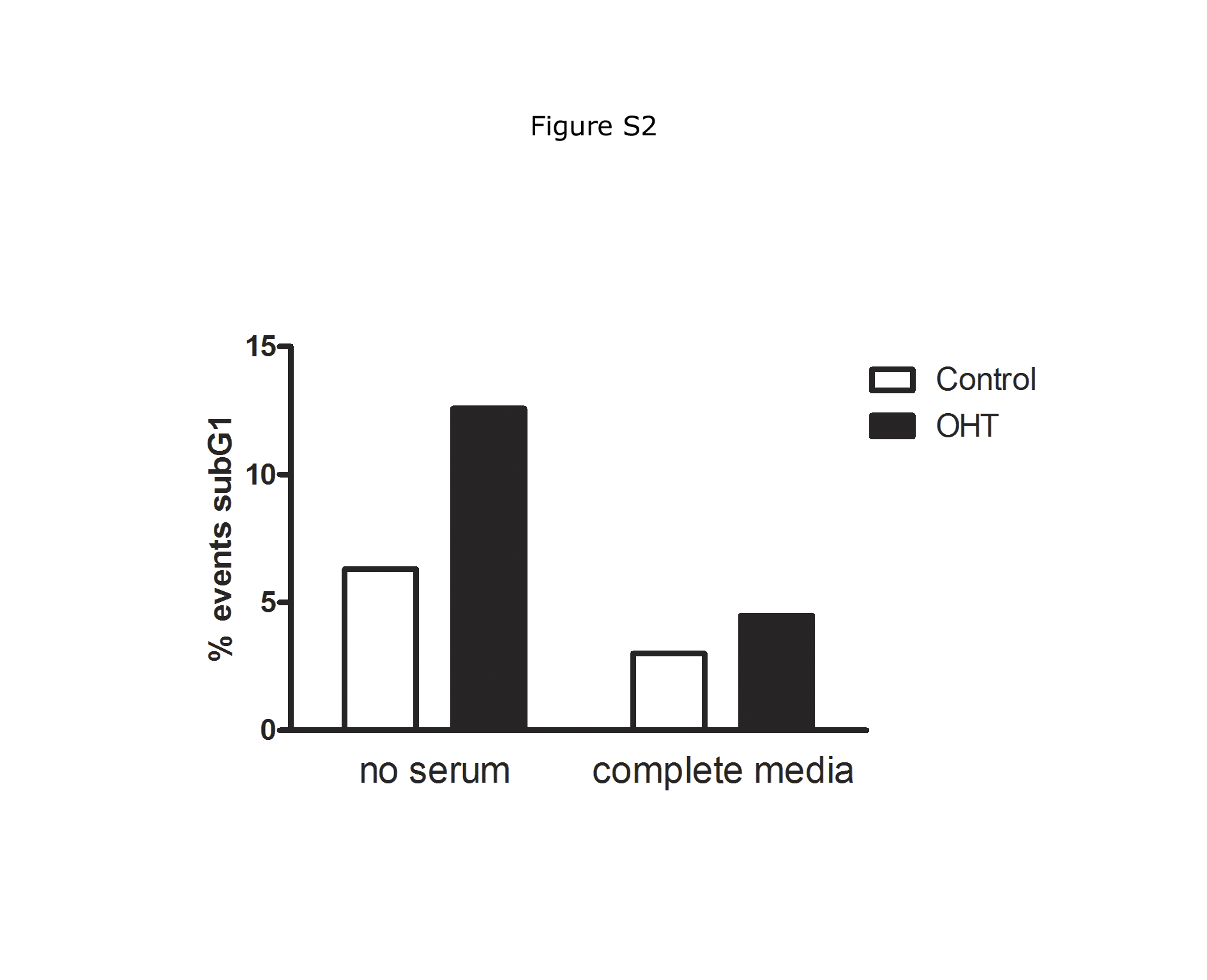

Supplement: Figure S2 — E2F1 induces the appearance of sub-diploid DNA peak. Stable ER-E2F1 PC12 cells were treated or not treated with OHT in the presence or in the absence of serum. Analysis of cell cycle was achieved by forward light scatter and was quantified the subdiploid DNA peak of cells at the indicated conditions. (TIF) [file pone.0051544.s002.tif]

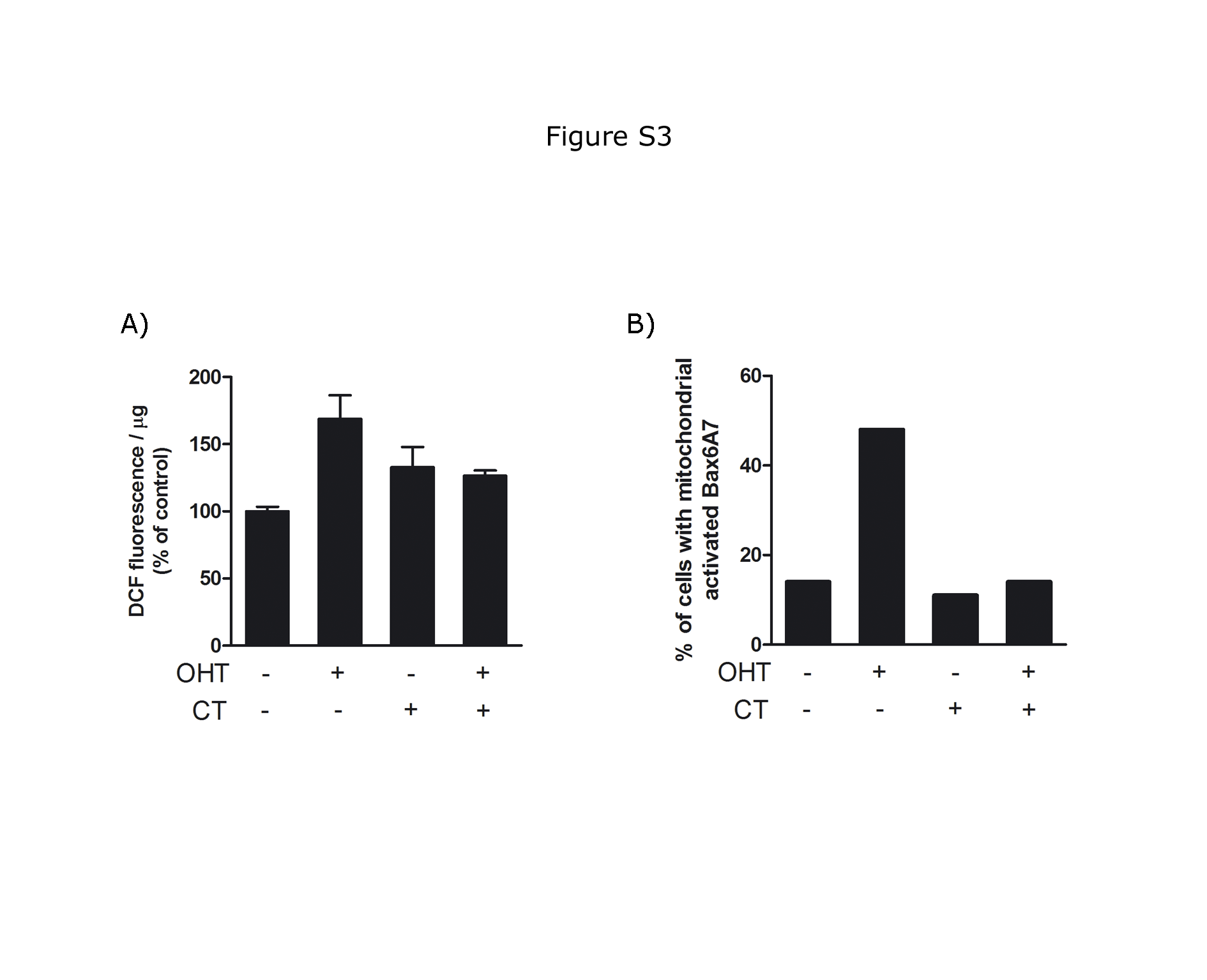

Supplement: Figure S3 — CT99021 inhibits ROS production and Bax activation induced by E2F1. (A) PC12 ER-E2F1 cells were serum-deprived and treated with (+) or without (−) OHT in the presence (+) or in the absence (−) of 10 µM CT99021 for 4 hours. ROS levels were analysed by using the oxidation-sensitive fluorescent probe H2DCFDA. Results are presented as Mean ± SEM, for n = 3. Student’s t-test value of ***p<0,0001 was considered statistically significant. (B) PC12 ER-E2F1 cells were serum-deprived and treated with (+) or without (−) OHT in the presence (+) or in the absence (−) of 10 µM CT99021 for 3 hours. Active Bax was detected by immunofluorescence using anti-Bax 6A7 clone antibody and anti-BAx 6A7 positives were quantified. (TIF) [file pone.0051544.s003.tif]
