# Supplementary material for: ROS Production Is Essential for the Apoptotic Function of E2F1 in Pheochromocytoma and Neuroblastoma Cell Lines
Source: PLoS One. 2012 Dec 12;7(12):e51544. doi: 10.1371/journal.pone.0051544 (PMC3520901; doi:10.1371/journal.pone.0051544)
Supplement: Table S1 — List of genes that are included on the Human Oxidative Stress and Antioxidant Defense RT2 Profiler PCR Array (Biosciences). (DOCX) [file pone.0051544.s004.docx]

**Table S1.**

| **Position** | **Unigene** | **GeneBank** | **Symbol** | **Description** | **Gene Name** |
| --- | --- | --- | --- | --- | --- |
| **A01** | Hs.418167 | NM_000477 | ALB | Albumin | DKFZp779N1935, PRO0883, PRO0903, PRO1341 |
| **A02** | Hs.654431 | NM_000697 | ALOX12 | Arachidonate 12-lipoxygenase | 12-LOX, 12S-LOX, LOG12 |
| **A03** | Hs.146559 | NM_021146 | ANGPTL7 | Angiopoietin-like 7 | AngX, CDT6, RP4-647M16.2, dJ647M16.1 |
| **A04** | Hs.406238 | NM_001159 | AOX1 | Aldehyde oxidase 1 | AO, AOH1 |
| **A05** | Hs.654439 | NM_000041 | APOE | Apolipoprotein E | AD2, LDLCQ5, LPG, MGC1571 |
| **A06** | Hs.125213 | NM_004045 | ATOX1 | ATX1 antioxidant protein 1 homolog (yeast) | ATX1, HAH1, MGC138453, MGC138455 |
| **A07** | Hs.144873 | NM_004052 | BNIP3 | BCL2/adenovirus E1B 19kDa interacting protein 3 | NIP3 |
| **A08** | Hs.502302 | NM_001752 | CAT | Catalase | MGC138422, MGC138424 |
| **A09** | Hs.514821 | NM_002985 | CCL5 | Chemokine (C-C motif) ligand 5 | D17S136E, MGC17164, RANTES, SCYA5, SISd, TCP228 |
| **A10** | Hs.502917 | NM_005125 | CCS | Copper chaperone for superoxide dismutase | MGC138260 |
| **A11** | Hs.69855 | NM_007158 | CSDE1 | Cold shock domain containing E1, RNA-binding | D1S155E, DKFZp779B0247, DKFZp779J1455, FLJ26882, RP5-1000E10.3, UNR |
| **A12** | Hs.513803 | NM_000101 | CYBA | Cytochrome b-245, alpha polypeptide | p22-PHOX |
| **B01** | Hs.95120 | NM_134268 | CYGB | Cytoglobin | HGB, STAP |
| **B02** | Hs.631770 | NM_001013742 | DGKK | Diacylglycerol kinase, kappa | - |
| **B03** | Hs.498727 | NM_014762 | DHCR24 | 24-dehydrocholesterol reductase | DCE, KIAA0018, Nbla03646, SELADIN1, seladin-1 |
| **B04** | Hs.272813 | NM_175940 | DUOX1 | Dual oxidase 1 | LNOX1, MGC138840, MGC138841, NOXEF1, THOX1 |
| **B05** | Hs.71377 | NM_014080 | DUOX2 | Dual oxidase 2 | LNOX2, NOXEF2, P138-TOX, TDH6, THOX2 |
| **B06** | Hs.171695 | NM_004417 | DUSP1 | Dual specificity phosphatase 1 | CL100, HVH1, MKP-1, MKP1, PTPN10 |
| **B07** | Hs.212088 | NM_001979 | EPHX2 | Epoxide hydrolase 2, cytoplasmic | CEH, SEH |
| **B08** | Hs.279259 | NM_000502 | EPX | Eosinophil peroxidase | EPO, EPP, EPX-PEN |
| **B09** | Hs.239 | NM_021953 | FOXM1 | Forkhead box M1 | FKHL16, FOXM1B, HFH-11, HFH11, HNF-3, INS-1, MPHOSPH2, MPP-2, MPP2, PIG29, TGT3, TRIDENT |
| **B10** | Hs.458283 | NM_197962 | GLRX2 | Glutaredoxin 2 | GRX2, bA101E13.1 |
| **B11** | Hs.333358 | NM_153002 | GPR156 | G protein-coupled receptor 156 | GABABL, MGC142261, PGR28 |
| **B12** | Hs.76686 | NM_000581 | GPX1 | Glutathione peroxidase 1 | GSHPX1, MGC14399, MGC88245 |
| **C01** | Hs.2704 | NM_002083 | GPX2 | Glutathione peroxidase 2 (gastrointestinal) | GI-GPx, GPRP, GSHPX-GI, GSHPx-2 |
| **C02** | Hs.386793 | NM_002084 | GPX3 | Glutathione peroxidase 3 (plasma) | GPx-P, GSHPx-3, GSHPx-P |
| **C03** | Hs.433951 | NM_002085 | GPX4 | Glutathione peroxidase 4 (phospholipid hydroperoxidase) | MCSP, PHGPx, snGPx, snPHGPx |
| **C04** | Hs.248129 | NM_001509 | GPX5 | Glutathione peroxidase 5 (epididymal androgen-related protein) | - |
| **C05** | Hs.448570 | NM_182701 | GPX6 | Glutathione peroxidase 6 (olfactory) | GPX5p, GPXP3, GPx-6, GSHPx-6, dJ1186N24, dJ1186N24.1 |
| **C06** | Hs.43728 | NM_015696 | GPX7 | Glutathione peroxidase 7 | CL683, FLJ14777, GPX6, GPx-7, GSHPx-7, NPGPx |
| **C07** | Hs.271510 | NM_000637 | GSR | Glutathione reductase | MGC78522 |
| **C08** | Hs.82327 | NM_000178 | GSS | Glutathione synthetase | GSHS, MGC14098 |
| **C09** | Hs.655292 | NM_001513 | GSTZ1 | Glutathione transferase zeta 1 | GSTZ1-1, MAAI, MAI, MGC2029 |
| **C10** | Hs.520459 | NM_001518 | GTF2I | General transcription factor IIi | BAP135, BTKAP1, DIWS, FLJ38776, FLJ56355, GTFII-I, IB291, SPIN, TFII-I, WBS, WBSCR6 |
| **C11** | Hs.80828 | NM_006121 | KRT1 | Keratin 1 | CK1, EHK, EHK1, EPPK, K1, KRT1A, NEPPK |
| **C12** | Hs.234742 | NM_006151 | LPO | Lactoperoxidase | MGC129990, MGC129991, SPO |
| **D01** | Hs.499674 | NM_000242 | MBL2 | Mannose-binding lectin (protein C) 2, soluble | COLEC1, HSMBPC, MBL, MBP, MBP-C, MBP1, MGC116832, MGC116833 |
| **D02** | Hs.191734 | NM_004528 | MGST3 | Microsomal glutathione S-transferase 3 | GST-III |
| **D03** | Hs.458272 | NM_000250 | MPO | Myeloperoxidase | - |
| **D04** | Hs.75659 | NM_002437 | MPV17 | MpV17 mitochondrial inner membrane protein | MTDPS6, SYM1 |
| **D05** | Hs.490981 | NM_012331 | MSRA | Methionine sulfoxide reductase A | PMSR |
| **D06** | Hs.73133 | NM_005954 | MT3 | Metallothionein 3 | GIF, GIFB, GRIF |
| **D07** | Hs.145932 | NM_004923 | MTL5 | Metallothionein-like 5, testis-specific (tesmin) | CXCDC2, MTLT, TESMIN |
| **D08** | Hs.647047 | NM_000265 | NCF1 | Neutrophil cytosolic factor 1 | FLJ79451, NCF1A, NOXO2, SH3PXD1A, p47phox |
| **D09** | Hs.587558 | NM_000433 | NCF2 | Neutrophil cytosolic factor 2 | FLJ93058, NCF-2, NOXA2, P67-PHOX, P67PHOX |
| **D10** | Hs.715767 | NM_003551 | NME5 | Non-metastatic cells 5, protein expressed in (nucleoside-diphosphate kinase) | NM23-H5, NM23H5, RSPH23 |
| **D11** | Hs.709191 | NM_000625 | NOS2 | Nitric oxide synthase 2, inducible | HEP-NOS, INOS, NOS, NOS2A |
| **D12** | Hs.657932 | NM_024505 | NOX5 | NADPH oxidase, EF-hand calcium binding domain 5 | MGC149776, MGC149777 |
| **E01** | Hs.534331 | NM_002452 | NUDT1 | Nudix (nucleoside diphosphate linked moiety X)-type motif 1 | MTH1 |
| **E02** | Hs.148778 | NM_181354 | OXR1 | Oxidation resistance 1 | FLJ10125, FLJ38829, FLJ40849, FLJ41673, FLJ42450, FLJ45656 |
| **E03** | Hs.475970 | NM_005109 | OXSR1 | Oxidative-stress responsive 1 | KIAA1101, OSR1 |
| **E04** | Hs.368525 | NM_020992 | PDLIM1 | PDZ and LIM domain 1 | CLIM1, CLP-36, CLP36, hCLIM1 |
| **E05** | Hs.146100 | NM_015553 | IPCEF1 | Interaction protein for cytohesin exchange factors 1 | KIAA0403, PIP3-E, RP3-402L9.2 |
| **E06** | Hs.78016 | NM_007254 | PNKP | Polynucleotide kinase 3'-phosphatase | EIEE10, MCSZ, PNK |
| **E07** | Hs.180909 | NM_002574 | PRDX1 | Peroxiredoxin 1 | MSP23, NKEFA, PAG, PAGA, PAGB, PRX1, PRXI, TDPX2 |
| **E08** | Hs.432121 | NM_005809 | PRDX2 | Peroxiredoxin 2 | MGC4104, NKEFB, PRP, PRX2, PRXII, TDPX1, TPX1, TSA |
| **E09** | Hs.523302 | NM_006793 | PRDX3 | Peroxiredoxin 3 | AOP-1, AOP1, MER5, MGC104387, MGC24293, PRO1748, SP-22 |
| **E10** | Hs.83383 | NM_006406 | PRDX4 | Peroxiredoxin 4 | AOE37-2, PRX-4 |
| **E11** | Hs.502823 | NM_181652 | PRDX5 | Peroxiredoxin 5 | ACR1, AOEB166, B166, MGC117264, MGC142283, MGC142285, PLP, PMP20, PRDX6, PRXV |
| **E12** | Hs.120 | NM_004905 | PRDX6 | Peroxiredoxin 6 | 1-Cys, AOP2, KIAA0106, MGC46173, NSGPx, PRX, aiPLA2, p29 |
| **F01** | Hs.153310 | NM_020820 | PREX1 | Phosphatidylinositol-3,4,5-trisphosphate-dependent Rac exchange factor 1 | KIAA1415, P-REX1 |
| **F02** | Hs.251386 | NM_006093 | PRG3 | Proteoglycan 3 | MBP2, MBPH, MGC126662, MGC141971 |
| **F03** | Hs.472010 | NM_183079 | PRNP | Prion protein | ASCR, CD230, CJD, GSS, MGC26679, PRIP, PrP, PrP27-30, PrP33-35C, PrPc, prion |
| **F04** | Hs.201978 | NM_000962 | PTGS1 | Prostaglandin-endoperoxide synthase 1 (prostaglandin G/H synthase and cyclooxygenase) | COX1, COX3, PCOX1, PGG, HS, PGHS-1, PGHS1, PHS1, PTGHS |
| **F05** | Hs.196384 | NM_000963 | PTGS2 | Prostaglandin-endoperoxide synthase 2 (prostaglandin G/H synthase and cyclooxygenase) | COX-2, COX2, GRIPGHS, PGG, HS, PGHS-2, PHS-2, hCox-2 |
| **F06** | Hs.332197 | NM_012293 | PXDN | Peroxidasin homolog (Drosophila) | D2S448, D2S448E, KIAA0230, MG50, PRG2, PXN, VPO |
| **F07** | Hs.444882 | NM_144651 | PXDNL | Peroxidasin homolog (Drosophila)-like | FLJ25471, VPO2 |
| **F08** | Hs.134623 | NM_014245 | RNF7 | Ring finger protein 7 | CKBBP1, ROC2, SAG |
| **F09** | Hs.128856 | NM_182826 | SCARA3 | Scavenger receptor class A, member 3 | APC7, CSR, CSR1, MSLR1, MSRL1 |
| **F10** | Hs.32148 | NM_203472 | SELS | Selenoprotein S | ADO15, MGC104346, MGC2553, SBBI8, SEPS1, VIMP |
| **F11** | Hs.275775 | NM_005410 | SEPP1 | Selenoprotein P, plasma, 1 | SELP, SeP |
| **F12** | Hs.253495 | NM_003019 | SFTPD | Surfactant protein D | COLEC7, PSP-D, SFTP4, SP-D |
| **G01** | Hs.472793 | NM_016276 | SGK2 | Serum/glucocorticoid regulated kinase 2 | H-SGK2, dJ138B7.2 |
| **G02** | Hs.466693 | NM_012237 | SIRT2 | Sirtuin 2 | FLJ35621, FLJ37491, SIR2, SIR2L, SIR2L2 |
| **G03** | Hs.443914 | NM_000454 | SOD1 | Superoxide dismutase 1, soluble | ALS, ALS1, IPOA, SOD, hSod1, homodimer |
| **G04** | Hs.487046 | NM_000636 | SOD2 | Superoxide dismutase 2, mitochondrial | IPOB, MNSOD, MVCD6 |
| **G05** | Hs.2420 | NM_003102 | SOD3 | Superoxide dismutase 3, extracellular | EC-SOD, MGC20077 |
| **G06** | Hs.516830 | NM_080725 | SRXN1 | Sulfiredoxin 1 | C20orf139, FLJ43353, Npn3, SRX1, YKL086W, dJ850E9.2 |
| **G07** | Hs.516807 | NM_006374 | STK25 | Serine/threonine kinase 25 | DKFZp686J1430, SOK1, YSK1 |
| **G08** | Hs.467554 | NM_000547 | TPO | Thyroid peroxidase | MSA, TDH2A, TPX |
| **G09** | Hs.134602 | NM_003319 | TTN | Titin | CMD1G, CMH9, CMPD4, DKFZp451N061, EOMFC, FLJ26020, FLJ26409, FLJ32040, FLJ34413, FLJ39564, FLJ43066, HMERF, LGMD2J, TMD |
| **G10** | Hs.98712 | NM_032243 | TXNDC2 | Thioredoxin domain containing 2 (spermatozoa) | DKFZp434H0311, MGC35026, SPTRX, SPTRX1 |
| **G11** | Hs.728817 | NM_003330 | TXNRD1 | Thioredoxin reductase 1 | GRIM-12, MGC9145, TR, TR1, TRXR1, TXNR |
| **G12** | Hs.443430 | NM_006440 | TXNRD2 | Thioredoxin reductase 2 | SELZ, TR, TR-BETA, TR3, TRXR2 |
| **H01** | Hs.534255 | NM_004048 | B2M | Beta-2-microglobulin | - |
| **H02** | Hs.412707 | NM_000194 | HPRT1 | Hypoxanthine phosphoribosyltransferase 1 | HGPRT, HPRT |
| **H03** | Hs.728776 | NM_012423 | RPL13A | Ribosomal protein L13a | L13A, TSTA1 |
| **H04** | Hs.592355 | NM_002046 | GAPDH | Glyceraldehyde-3-phosphate dehydrogenase | G3PD, GAPD, MGC88685 |
| **H05** | Hs.520640 | NM_001101 | ACTB | Actin, beta | PS1TP5BP1 |
| **H06** | N/A | SA_00105 | HGDC | Human Genomic DNA Contamination | HIGX1A |
| **H07** | N/A | SA_00104 | RTC | Reverse Transcription Control | RTC |
| **H08** | N/A | SA_00104 | RTC | Reverse Transcription Control | RTC |
| **H09** | N/A | SA_00104 | RTC | Reverse Transcription Control | RTC |
| **H10** | N/A | SA_00103 | PPC | Positive PCR Control | PPC |
| **H11** | N/A | SA_00103 | PPC | Positive PCR Control | PPC |
| **H12** | N/A | SA_00103 | PPC | Positive PCR Control | PPC |
