# Supplementary material for: ROS Production Is Essential for the Apoptotic Function of E2F1 in Pheochromocytoma and Neuroblastoma Cell Lines
Source: PLoS One. 2012 Dec 12;7(12):e51544. doi: 10.1371/journal.pone.0051544 (PMC3520901; doi:10.1371/journal.pone.0051544)
Supplement: Table S2 — mRNA changes after OHT addition in SK-N-JD cells.- RT2Profiler human oxidative stress and antioxidant defense PCR Arrays (Bioscience) were performed according to the manufacture’s protocols. Expression levels were compared between with and without OHT addition. Hypoxanthine phosphoribosyltransferase 1 gene was used as control for each gene expression calculation, and the extent of change in the expression of each gene was calculated by the ΔCt method. We show the genes that were indicated on Table1. (DOCX) [file pone.0051544.s005.docx]

**Table S2**.

| *Unigen* | *Symbol* | *Description* | *Fold Change* |
| --- | --- | --- | --- |
|  |  |  | *SK-N-JD (OHT/control)* |
| Hs.95120 | *CYGB* | Cytoglobin | 9.43 |
| Hs.502823 | *PRDX5* | Peroxiredoxin 5 | 0.92 |
| Hs.502917 | *CCS* | Copper chaperone for superoxide dismutase | 0.94 |
| Hs.728817 | *TXNRD1* | Thioredoxin reductase 1 | 0.96 |
| Hs.134602 | *TTN* | Titin | 1.23 |
| Hs.443430 | *TXNRD2* | Thioredoxin reductase 2 | 1.06 |
| Hs.251386 | *PRG3* | Proteoglycan 3 | 0.22 |
| Hs.234742 | *LPO* | Lactoperoxidase | 0.24 |
| Hs.631770 | *DGKK* | Diacylglycerol kinase, kappa | 2.67 |
| Hs.239 | *FOXM1* | Forkhead box M1 | 1.10 |
| Hs.146559 | *ANGPTL7* | Angiopoietin-like 7 | 0.72 |
| Hs.654439 | *APOE* | Apolipoprotein E | 1.71 |
| Hs.128856 | *SCARA3* | Scavenger receptor class A, member 3 | 1.28 |
| Hs.180909 | *PRDX1* | Peroxiredoxin 1 | 0.96 |
| Hs.467554 | *TPO* | Thyroid peroxidase | 1.38 |
| *Genes upregulated* | |  |  |
| Hs.333358 | *GPR156* | G protein-coupled receptor 156 | 1.34 |
| Hs.71377 | *DUOX2* | Dual oxidase 2 | 0.81 |
